# Fine-mapping and cross-validation of QTLs linked to fatty acid composition in multiple independent interspecific crosses of oil palm

**DOI:** 10.1186/s12864-016-2607-4

**Published:** 2016-04-14

**Authors:** Ngoot-Chin Ting, Zulkifli Yaakub, Katialisa Kamaruddin, Sean Mayes, Festo Massawe, Ravigadevi Sambanthamurthi, Johannes Jansen, Leslie Eng Ti Low, Maizura Ithnin, Ahmad Kushairi, Xaviar Arulandoo, Rozana Rosli, Kuang-Lim Chan, Nadzirah Amiruddin, Kandha Sritharan, Chin Ching Lim, Rajanaidu Nookiah, Mohd Din Amiruddin, Rajinder Singh

**Affiliations:** Malaysian Palm Oil Board (MPOB), P.O. Box 10620, 50720 Kuala Lumpur, Malaysia; Plant and Crop Sciences, Sutton Bonington Campus, University of Nottingham, Sutton Bonington, Loughborough, LE12 5RD UK; School of Biosciences, University of Nottingham Malaysia Campus, Jalan Broga, 43500 Semenyih, Selangor Malaysia; Biometris, Wageningen University and Research Centre, P.O. Box 100, 6700 AC Wageningen, The Netherlands; United Plantations Bhd., Jendarata Estate, 36009 Teluk Intan, Perak Malaysia

**Keywords:** *Elaeis guineensis*, *Elaeis oleifera*, Backcross-two (BC_2_), Iodine value (IV), Palmitic acid (C16:0), Oleic acid (C18:1)

## Abstract

**Background:**

The commercial oil palm (*Elaeis guineensis* Jacq.) produces a mesocarp oil (commonly called ‘palm oil’) with approximately equal proportions of saturated and unsaturated fatty acids (FAs). An increase in unsaturated FAs content or iodine value (IV) as a measure of the degree of unsaturation would help to open up new markets for the oil. One way to manipulate the fatty acid composition (FAC) in palm oil is through introgression of favourable alleles from the American oil palm, *E. oleifera*, which has a more unsaturated oil.

**Results:**

In this study, a segregating *E. oleifera* x *E. guineensis* (OxG) hybrid population for FAC is used to identify quantitative trait loci (QTLs) linked to IV and various FAs. QTL analysis revealed 10 major and two putative QTLs for IV and six FAs, C14:0, C16:0, C16:1, C18:0, C18:1 and C18:2 distributed across six linkage groups (LGs), OT1, T2, T3, OT4, OT6 and T9. The major QTLs for IV and C16:0 on LGOT1 explained 60.0 – 69.0 % of the phenotypic trait variation and were validated in two independent BC_2_ populations. The genomic interval contains several key structural genes in the FA and oil biosynthesis pathways such as *PATE/FATB*, *HIBCH*, *BASS2*, *LACS4* and *DGAT1* and also a relevant transcription factor (TF), *WRI1*. The literature suggests that some of these genes can exhibit pleiotropic effects in the regulatory networks of these traits. Using the whole genome sequence data, markers tightly linked to the candidate genes were also developed. Clustering trait values according to the allelic forms of these candidate markers revealed significant differences in the IV and FAs of the palms in the mapping and validation crosses.

**Conclusions:**

The candidate gene approach described and exploited here is useful to identify the potential causal genes linked to FAC and can be adopted for marker-assisted selection (MAS) in oil palm.

**Electronic supplementary material:**

The online version of this article (doi:10.1186/s12864-016-2607-4) contains supplementary material, which is available to authorized users.

## Background

The African oil palm (*Elaeis guineensis* Jacq.) is the major oil crop in the world today [1, 2]. The wide range of applications (80.0–85.0 %) for mesocarp oil is due to its FAC which is suitable for making common consumable products (e.g. cooking oil, butters and margarine), pharmaceuticals and animal feedstocks. In addition, palm oil has industrial applications, e.g. making biodiesel, oleochemicals, cosmetics and textiles.

Palm oil has roughly equal proportions of saturated and unsaturated FAs. The saturated FAs are palmitic (C16:0, 44.0 %), stearic (C18:0, 4.5 %), myristic (C14:0, 1.1 %), arachidic (C20:0, 0.3 %) and lauric (C12:0, 0.2 %). The unsaturated FAs include 39.2 % oleic (C18:1), 10.1 % linoleic (C18:2), 0.3 % linolenic (C18:3) and 0.1 % palmitoleic (C16:1) [3, 4]. In comparison, the mesocarp oil from the American oil palm, *E. oleifera*, is much more unsaturated with, 58.0–68.0 % C18:1, 14.0–20.0 % C18:2 and only 15.0–20.0 % C16:0 and 0.4–1.5 % C18:0, as observed in the MPOB Colombian germplasm collection [5]. As the current world demand is for less saturated edible oils, it would be advantageous if the *E. guineensis* oil can be selected to have a composition closer to *E. oleifera* oil [6, 7].

The desirable FAC in Colombian *E. oleifera* oil makes the palm an ideal material for introgression into elite *E. guineensis* such as the MPOB Nigerian germplasm (T128), which is already known for its higher unsaturated oil content [8, 9]. The T128 germplasm has been distributed as a high IV material [MPOB’s PORIM Series 2 (PS2)] and extensively used in various interspecific breeding programs by the oil palm industry [9, 10]. Therefore, it is important to capture the favourable alleles linked to high IV in the successive hybrids and backcrosses. The resulting *E. oleifera* × *E. guineensis* interspecific hybrid population was found to be segregating for IV and major FA traits which allowed for identification of QTLs linked to these traits. A number of QTLs for IV and FAC located on the T128 parental genetic map and mostly flanked by amplified fragment length polymorphism (AFLP) and restricted fragment length polymorphism (RFLP) markers were reported by Singh *et al*. [8]. The current study reports an extension of the work initiated by Singh et al. [8] by identifying QTLs on a higher density simple sequence repeat (SSR) and single nucleotide polymorphism (SNP)-based genetic map [11].

In plants, the FA and triacylglycerol (TAG) biosynthesis pathways occur in separate compartments. *De novo* FA synthesis occurs in the plastid and the growing FA chain is held by acyl carrier protein (ACP). Subsequently, acyl-ACPs are hydrolysed by acyl-ACP thioesterases and the resulting non-esterified FAs exported to the endoplasmic reticulum (ER) for assembly into TAGs [12, 13]. Recently, oil palm transcriptome data from developing fruits (particularly from the mesocarp tissues) were used to investigate the regulatory mechanisms of genes and transcription factors (TFs) governing the synthesis of FA and TAG [14, 15]. The formation of FA destined for oil accumulation starts around 110 days after pollination (DAP) and reaches its peak at 120 DAP. It is during this period that TAGs begin to accumulate in the mesocarp and reach a peak at 160 DAP [14].

The transcriptome data have also opened up new avenues to develop candidate markers for FA biosynthesis genes with oil palm orthologues identified for β-ketoacyl-ACP synthases (*KASI* and *II*), acyl-ACP thioesterases (*FATA* and *B*) and stearoyl-ACP desaturase (*SAD*). The data was exploited by Montoya et al. [16], where SNP markers were developed from these candidate genes and 14 of them were polymorphic and located on the *E. oleifera* × *E. guineensis* pseudo-backcross-one (BC_1_) genetic map. Among the 14 SNP markers, four located within the confidence intervals of QTLs linked to IV and FAC [16, 17]. Taking a slightly different approach, potential candidate genes and a TF associated with biosynthesis of FA and TAG were identified in the major QTL regions revealed in this study. This was done by comparing the QTL regions (linked to FAC) to the oil palm genome assembly [18]. Markers based on these candidate genes were developed to saturate the QTL intervals. The saturated QTL regions revealed closely linked markers and, if validated across different genetic backgrounds, these markers could have utility in a MAS program. A similar approach has been applied with great success in rice and even oil palm, in identifying candidate genes linked to mapped QTLs [19, 20].

The second part of this study focused on validating the consistency of QTLs linked to IV and FAC in two independent pseudo-BC_2_ populations of *E. guineensis* × *E. oleifera* (GxO). The validation families were derived from different genetic backgrounds compared to the populations described by Montoya et al. [16, 17]. In addition to validating some of the previously reported QTL locations, this study revealed additional genomic regions influencing IV and FAC. Compilation of different favourable alleles of QTLs obtained from various genetic backgrounds will help to develop effective strategies for the application of MAS in an interspecific hybrid breeding program. In soybean, Wang et al. [21] described MAS for C16:0 and C18:0 through pyramiding of two to three QTLs, detected across various genetic backgrounds, exhibiting significant cumulative effects. Similarly, this study aimed to uncover a set of markers that can be used to select for favourable alleles linked to unsaturation, at least in the genetic backgrounds examined.

## Methods

### Mapping population

The mapping population ‘OxG’ is an interspecific cross between a Colombian *E. oleifera* (UP1026, maternal parent) and an Nigerian *E. guineensis tenera* (T128, paternal parent) as reported by Singh *et al*. [8] and Ting *et al*. [11]. The population consists of 118 hybrids and was created and is maintained by United Plantations Berhad (UPB), Perak, Malaysia. Of these, eight palms were excluded from the linkage analysis due to relatively high recombination frequencies [8]. An additional two palms died and therefore, only 108 palms were used in this study.

### Validation crosses

**Fig. 1:**
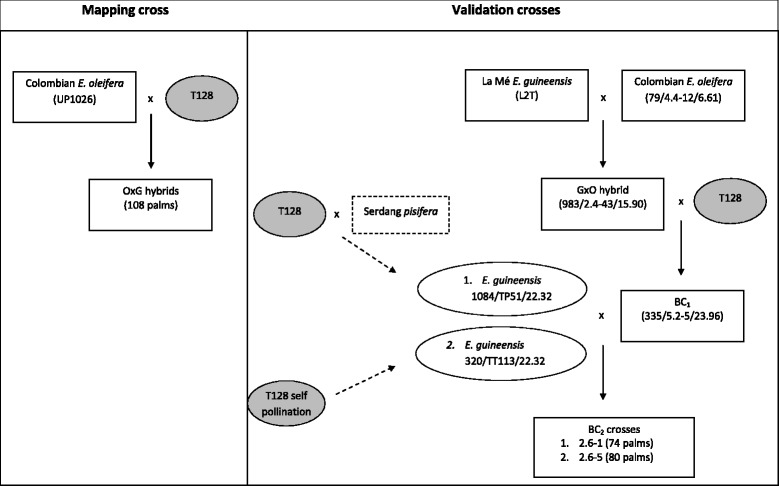
The OxG mapping population and BC_2_ validation crosses used in this study. The 108 OxG interpecific hybrids (left) were created by crossing a maternal Colombian *E. oleifera* (UP1026) with a paternal palm T128 (*tenera*), from the Nigerian germplasm. The same T128 palm was also used in creating the BC_2_ validation crosses (right). The La Mé *E. guineensis* (L2T, maternal parent) was crossed with a Colombian *E. oleifera* (79/4.4–12/6.61, paternal parent) and the resulting GxO F_1_ hybrid (983/2.4–43/15.90) was then backcrossed with the T128 palm to produce the BC_1_. Pollen from a selected BC_1_ palm (335/5.2–5/23.96) was used to cross-pollinate two *E. guineensis* palms. The first *E. guineensis* (1084/TP51/22.32) was derived from a cross between the T128 and a Serdang *pisifera* and the second palm (320/TT113/22.32) was derived from a self-pollination of the T128 palm. The two BC_2_ families namely, 2.6–1 and 2.6–5 consisted of 74 and 80 palms, respectively

Two BC_2_ crosses from an independent breeding program at UPB were used to attempt to validate the QTLs detected in the OxG mapping population. The breeding approach used in creating the BC_2_ crosses is illustrated in Fig. 1. The first cross was between a La Mé *E. guineensis* (L2T, maternal parent) and a Colombian *E. oleifera* (79/4.4–12/6.61, paternal parent) and is termed ‘GxO’. The resulting GxO F_1_ hybrid (983/2.4–43/15.90) was then backcrossed with palm T128, from the Nigerian germplasm to produce the BC_1_. Pollen was then obtained from a selected BC_1_ palm (335/5.2–5/23.96) to cross-pollinate two *E. guineensis* palms. The first *E. guineensis* (1084/TP51/22.32) was derived from a cross between the palm T128 and a Serdang *pisifera* and the second palm (320/TT113/22.32) was derived from a self-pollination of the original T128 palm. The two BC_2_ families are named Progenies ‘2.6–1’ and ‘2.6–5’, consisting of 74 and 80 palms, respectively. They were field planted in the year 2000. The female parent of 2.6–5 (320/TT113/22.32) died before any leaf and fruit could be sampled. Therefore, two siblings to 320/TT113/22.32 were genotyped to help with scoring and phase configuration of the markers used for the construction of genetic maps.

### Extraction and analysis of palm oil

The procedures for sampling ripe fruits and extracting mesocarp oil were as described by Singh et al. [8]. The PORIM Test Method [22] was applied to measure the IV and FAC in the palm oil.

### Candidate SNP markers and genetic linkage map construction

Candidate SNP markers (designated SNPE) flanking various genes associated with FA and oil biosynthesis were mined from the P5 genome build. The oil palm SNP assay design and genotyping were performed by a service provider, Agena Bioscience, Inc. (San Deigo, California) using the iPLEX ^®^ biochemistry on MassArray® system [23]. A custom two-multiplexed genotyping assay was designed and optimized for a panel of 40 SNPs using the Assay Design Suite 1.0 software (Agena Bioscience, Inc. San Deigo, California). Primary PCR primers (forward and reverse) were designed to contain a common 5’ 10-mer tag (ACGTTGGATG) and synthesized by IDT (Singapore and USA). The primary PCR reaction was carried out to amplify DNA fragments of approximately 100 bp containing targeted SNPs from the good quality (A_260_/A_280_ ratio >1.7) oil palm genomic DNA (20 ng/μl). The PCR conditions included an initial denaturation at 95 °C for 2 min, 45 cycles of 95 °C denaturation (20 s), 56 °C annealing (30 s) and 72 °C extension (1 min), and a final extension at 72 °C for 5 min. The remaining unincorporated dNTPs in the PCR product was inactivated using shrimp alkaline phosphatase (SAP) treatment (Agena Bioscience, Inc. San Deigo, California). This was followed by an allele-specific single base primer extension reaction performed using the iPLEX Gold Reaction Kit (Agena Bioscience, Inc. San Deigo, California). Reaction cocktail containing extend primer, buffer, enzyme, and mass-modified ddNTPs (prepared following the manufacturer’s protocol) was added to the primary PCR product and proceeded to extension reaction at 94 °C initial denaturation (30 s), 40 cycles of 95 °C denaturation (5 s), 52 °C annealing (5 s, 5 cycles) and 80 °C extension (5 s, 5 cycles), and a final extension at 72 °C for 3 min. The extension reaction product was desalted using the SpectroCLEAN (Agena Bioscience, Inc. San Deigo, California) resin treatment and transferred onto the SpectroCHIP® Array (Agena Bioscience, Inc. San Deigo, California) for data analysis using Matrix-Assisted Laser Desorption/Ionization Time-Of-Flight (MALDI-TOF) mass spectrometry. The SNP genotyping data and report were generated using the Typer 4.0 software (Agena Bioscience, Inc. San Deigo, California). The genotyping data were incorporated into the existing dataset [11] for construction of the genetic map.

Furthermore, the AFLP and RFLP markers reported by Singh et al. [8] to be flanking the QTL regions were also incorporated into the existing linkage map which was re-constructed using JoinMap® 4.1 [24] as described previously [11]. The genetic maps for the two validation crosses, 2.6–1 and 2.6–5, were similarly constructed using the same set of SNP and SSR markers (except SNPE markers). Comparison of the genetic maps for OxG, 2.6–1 and 2.6–5 was carried out using MapChart 2.2 [25].

### Quantitative trait loci (QTL) analyses

In the OxG population, the QTL analysis was performed using Interval Mapping (IM), the Multiple-QTL Model (MQM) and the Kruskal-Wallis non-parametric ranking tests (KW) with the default parameters in MapQTL 6 [26]. The 95 % genome-wide LOD significance threshold for each trait was determined by the permutation test option, with 1,000 permutations. Each candidate QTL interval was further analyzed using G model (GM) [27]. The GM analysis first estimated the genome-wide background effects of markers (co-factors) from all the LGs (except the LG being analyzed) using a random effects model (ridge-regression best linear unbiased prediction, RR-BLUP). The background effects were used to correct and determine significant markers by backward elimination (screening for QTL) on each LG. Finally, the effect of each significant marker was estimated using the multiple regression coefficients. Similar QTL analysis approaches were also applied in the 2.6–1 and 2.6–5 crosses to validate the detected QTLs.

### Development of candidate SSR markers within QTL confidence regions

Development of SSR markers (with nomenclature sPSc) for candidate FA genes and TF was carried out by aligning contigs and clone sequences (http://genomsawit.mpob.gov.my) containing the SNPs and SSRs mapped in the QTL confidence intervals, using BLASTN [28]. Markers from another independent genetic map (LGDP1) [11] that localize in the QTL-syntenic regions were also included in the similarity search. Scaffold regions linked to QTL confidence intervals were extracted from the P5 genome build and searched for sequence similarity (BLASTN and BLASTX) against the NCBI databases (nt, nr and refseq_protein). Sequences with significant similarity (BLASTN e-value of < 1e-25 and 95 % identity over total sequence length) to genes and TFs associated with FA and TAG syntheses were selected to attempt to mine SSRs using the MIcroSAtellite identification tool (MISA) [29].

Four percent Super Fine Resolution (SFR) agarose (Amresco, USA) gel electrophoresis was used to screen for potential polymorphism of the candidate SSRs against a panel of six randomly selected OxG hybrids and the two parental palms. The informative SSRs were then PCR-amplified using the M13-tailed primer (5’CACGACGTTGTAAAACGAC3’) approach as described by Ting et al. [30]. Fragment analysis of the PCR products was performed by a service provider at the Centre for Marker Discovery and Validation (CMDV), Malaysia using an ABI 3730XL DNA analyzer (Applied Biosystems, USA) with fluorescent dye sets comprising FAM, NED, PET, VIC and GeneScan™-500 LIZ^®^ Size Standard. Sizing of the SSR alleles was performed using the GeneMapper® 4.1 software (Applied Biosystems, USA) and genotype scoring was as described previously [11, 30].

## Results

### Mapping candidate SNPs for FA genes onto the OxG genetic map

In the OxG interspecific population, the existing paternal T128 (T) and partially integrated (OT) maps had 1,121 markers/16 LGs/1,759 cM and 899 markers/10 LGs/1,249 cM, respectively [11]. The two genetic maps were constructed mainly with SNP and SSR markers replacing the traditional AFLP and RFLP markers used in the earlier version (252 markers/21 LGs/1,815 cM) [8]. The new marker systems are technically more user-friendly, cheaper and give better reproducibility than the AFLP and RFLP markers. However, to anchor the previously [8] detected QTLs, the AFLP and RFLP markers flanking each QTL interval were also incorporated into this study.

An additional 40 SNPs (SNPE) were identified flanking various FA genes distributed across 22 scaffolds of the P5 genome build. Of these, seven for palmitoyl-ACP thioesterase (*PATE/FATB* – SNPE00431), oleoyl-CoA desaturase (*FAD2* – SNPE00437), linoleoyl-CoA desaturase (*FAD3* – SNPE00401), enoyl-ACP reductase (*ENR1* – SNPE00415 and 00416) and stearoyl-ACP desaturase (*SAD* – SNPE00427 and 00434) – were polymorphic in the OxG mapping population. These candidate SNP markers were mapped onto LGs OT1, T2, OT11, OT12 and T14 (Additional file 1) and used in the subsequent QTL analysis. For the OxG mapping population, as only 10 integrated LGs (OT 1, 4, 6, 7, 8, 10, 11, 12, 13 and 15) were available, six LGs (T2, 3, 5, 9, 14 and 16) from the T128 paternal map were also included in the QTL analysis.

### BC_2_ genetic linkage maps

In the BC_2_ validation crosses, two independent integrated maps with 1,755 markers/16 LGs/1,499.5 cM (unpublished) and 1,184 markers/18 LGs/1,589.7 cM [31] were constructed for the 2.6–1 and 2.6–5 populations, respectively. Resolution of the two genetic maps was good and the linear order of markers comparable to that in OxG with an average gap of 0.9 cM (2.6–1) and 1.3 cM (2.6–5). Both the high density genetic maps were used for QTL analysis.

### IV and FAC quantitative phenotypic data

In the OxG cross, palm oil was obtained from 85 F_1_ palms and measured for IV, C14:0, C16:0, C16:1, C18:0, C18:1, C18:2 and C18:3. The remaining palms could not be sampled due to sterility issues, abortive bunches and crown disease. Also 13 palms had died before phenotyping. The present phenotypic data set includes an additional four palms to that reported by Singh et al. [8] and is summarised in Table 1. Overall, the distribution of phenotypic data was similar to that reported previously. The IV (*p* = 0.20) and major FAs, C16:0 (*p* = 0.05), C18:1 (*p* = 0.16) and C18:2 (*p* = 0.20) showed a normal distribution (Kolmogorov-Smirnov, SPSS 16.0). However, the four minor components, C14:0 (*p* = 0.0), C16:1 (*p* = 0.0), C18:0 (*p* = 0.02) and C18:3 (*p* = 0.0) did not follow a normal distribution, also previously observed by Montoya et al. [16]. For C18:0, the data was converted to a normal distribution (*p* = 0.20) by log_10_ transformation, but the approach did not convert C14:0, C16:1 and C18:3 to a normal distribution. For these non-normally distributed traits, KW analysis was used. Correlation analysis gave similar results to those by Singh et al. [8] (Additional file 2).

**Table 1:** Summary of phenotypic data in the OxG mapping population and two BC_2_ (2.6-1 and 2.6–5) validation crosses

Trait	OxG (*n* = 85)			2.6–1 (*n* = 54)			2.6–5 (*n* = 57)		
	Mean (% ± SD)	Variance	Range (%)	Mean (% ± SD)	Variance	Range (%)	Mean (% ± SD)	Variance	Range (%)
Iodine value (IV)	70.98 (±2.88)	8.28	65.25–77.33	65.13 (±2.55)	6.51	60.19–69.86	63.44 (±3.24)	10.46	57.12–71.60
Myristic acid (C14:0)	0.29 (±0.09)	0.01	0.14–0.55	0.27 (±0.08)	0.007	0.14–0.49	0.43 (±0.14)	0.02	0.17–0.75
Palmitic acid (C16:0)	29.22 (±3.07)	9.45	22.25–34.33	31.06 (±0.08)	6.08	24.73–36.68	35.29 (±2.86)	8.15	26.85–41.69
Palmitoleic acid (C16:1)	0.43 (±0.14)	0.02	0.20–0.83	0.12 (±0.02)	0.000	0.07–0.16	0.18 (±0.06)	0.003	0.08–0.34
Stearic acid (C18:0)	2.08 (±0.32)	0.10	1.50–3.10	6.15 (±1.31)	1.73	3.29–9.43	3.79 (±0.93)	0.86	2.11–6.48
Oleic acid (C18:1)	53.96 (±3.19)	10.20	48.20–61.45	48.61 (±2.87)	8.25	40.92–57.18	47.08 (±3.68)	13.55	37.58–54.48
Linoleic acid (C18:2)	12.76 (±0.91)	0.82	10.45–15.15	12.86 (±1.41)	1.98	9.60–16.29	12.67 (±2.07)	4.27	8.15–17.65
Linolenic acid (C18:3)	0.50 (±0.08)	0.01	0.40–0.65	0.36 (±0.06)	0.004	0.16–0.49	0.22 (±0.15)	0.02	0–0.53

Means (%), ranges and variances measured for iodine value and various fatty acid contents in palm oil

For the two BC_2_ crosses, data were collected for 54 and 57 palms of the 2.6–1 and 2.6–5 crosses, respectively. The other palms did not bear any fruit or had died before sampling could be performed. In 2.6–1, the widest data range observed (Table 1) was for C18:1 (40.9–57.2 % ± 2.9) and C16:0 (24.7–36.7 % ± 0.08). All the data showed normal distributions except for IV, C14:0 and C16:1. However, log_10_ transformation (IV) and the discarding of one or two outliers (for C14:0 and C16:1) returned the distributions to normality. Outliers were identified by using a Boxplot and comparing the observed and expected mean (5 % trimmed mean, SPSS 16.0) values. Significant positive correlations were observed between C14:0 and C16:0 (*r* = 0.72), IV and C18:1 (*r* = 0.51) and, IV and C18:2 (*r* = 0.51). Strong negative correlations (*r* = − 0.62 to–0.75) were observed for C16:0 and C18:1, IV and C16:0, IV and C14:0 and, C16:1 and C18:0 while, C14:0 and C18:2, C14:0 and C18:1 and, C18:1 and C18:2 showed moderate negative correlations of −0.38 to –0.48 (Additional file 3).

In 2.6–5, C18:1 and C16:0 also showed the widest data distribution of 37.6–54.5 % (SD = 3.7) and 26.9–41.7 % (SD = 2.9), respectively. The phenotypic data (except for C14:0, C16:1 and C18:3) demonstrated a normal distribution. For C14:0 and C16:1, normality was improved after removing one outlier. The phenotypes IV and C18:2, C14:0 and C16:0, C14:0 and C18:3 and, C14:0 and C16:1, showed moderate correlations (*r* = 0.42–0.55) while, negative correlations were obtained for IV and C16:0, C16:0 and C18:1, C18:1 and C18:2, C14:0 and C18:1 and, C16:1 and C18:0 (Additional file 4).

### OxG: QTLs linked to IV and FAC

This study aimed to identify the QTLs linked to IV and FAC in the improved SNP and SSR-genetic map of the OxG cross [11]. Ten genome-wide significant QTLs and two putative QTLs (at chromosome-wide threshold levels) linked to IV, C14:0, C16:0, C16:1, C18:0, C18:1 and C18:2 were identified on LGs OT1, T2, T3, OT4, OT6 and T9 using a combined QTL detection method – IM, MQM and KW. Only markers or regions consistently linked to the specific QTL using all three approaches were considered to be significant QTL. Subsequently, GM was used to estimate the effects of closely flanked markers using multiple regression coefficients after backward elimination and adjustment for the background marker effects [27].

**Fig. 2:**
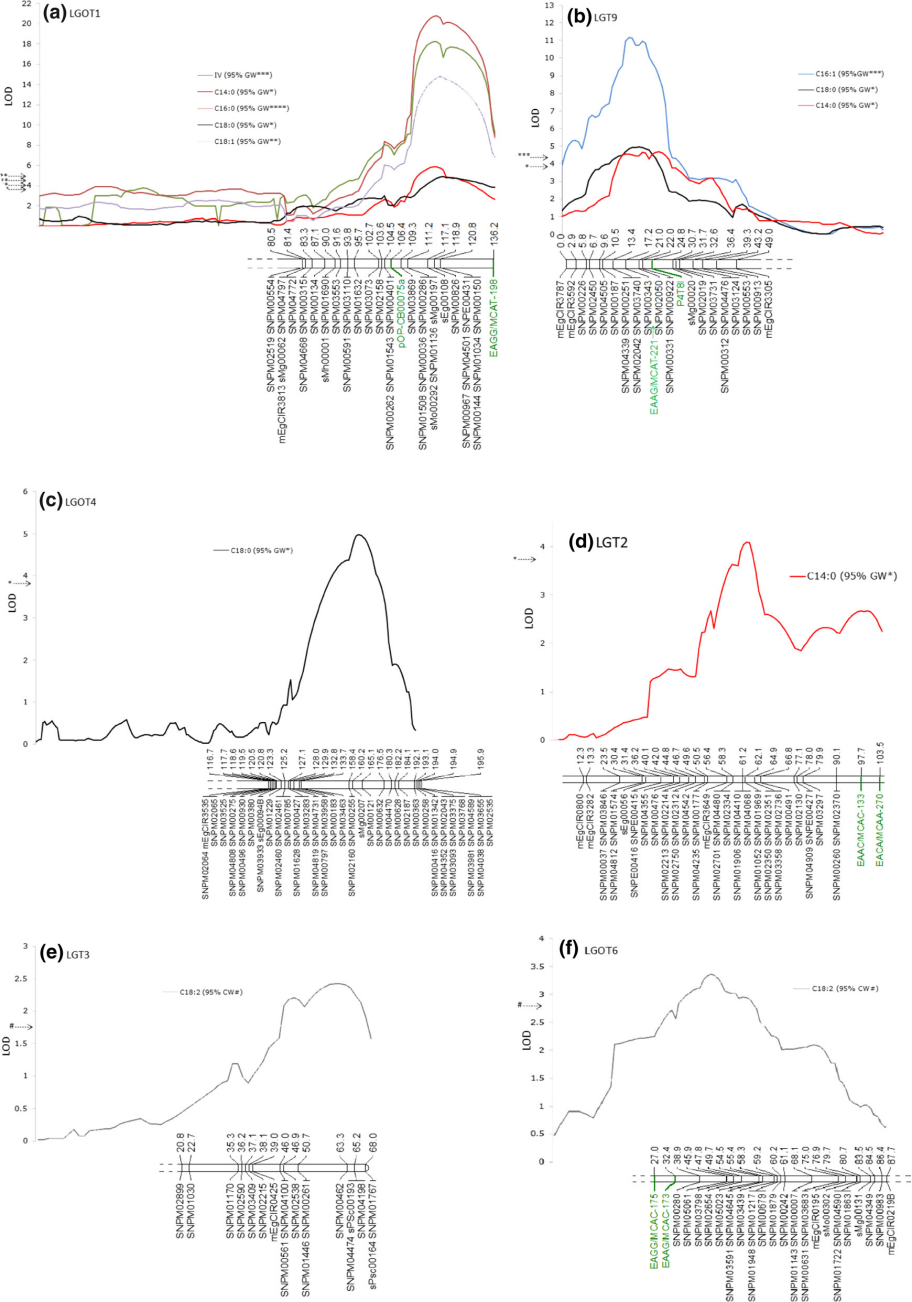
QTL profiles linked to IV and FAC on the improved genetic linkage map of OxG. The graphs (**a** – **f**) present QTLs (represented by different colour lines) significant at 95 % genome-wide (GW) and chromosome-wide (CW) thresholds in linkage groups (LGs) OT1, T9, OT4, T2, T3 and OT6

On the improved LGOT1 (labelled Group 1 previously), three major QTLs (for IV, C16:0 and C18:1) and two minor QTLs (for C14:0 and C18:0) were revealed in the same map interval previously reported between markers pOP-CB00075a and EAGG/MCAT-198 (Fig. 2, a). On the current LGOT1, the significance interval between the two markers (a 12 cM gap was reported previously) was filled by 12 SNPs and three SSRs. Of these, six SNP markers, including a *PATE* candidate SNP (SNPM04501, SNPM00967, SNPM00144, SNPM01034, SNPM00150 and SNPE00431), were within the QTL confidence interval (Fig. 2). This genomic region was of interest and further investigation was carried out with the additional results presented below in the ‘*Fine-mapping of the QTLs on LGOT1*’ section.

**Fig. 3:**
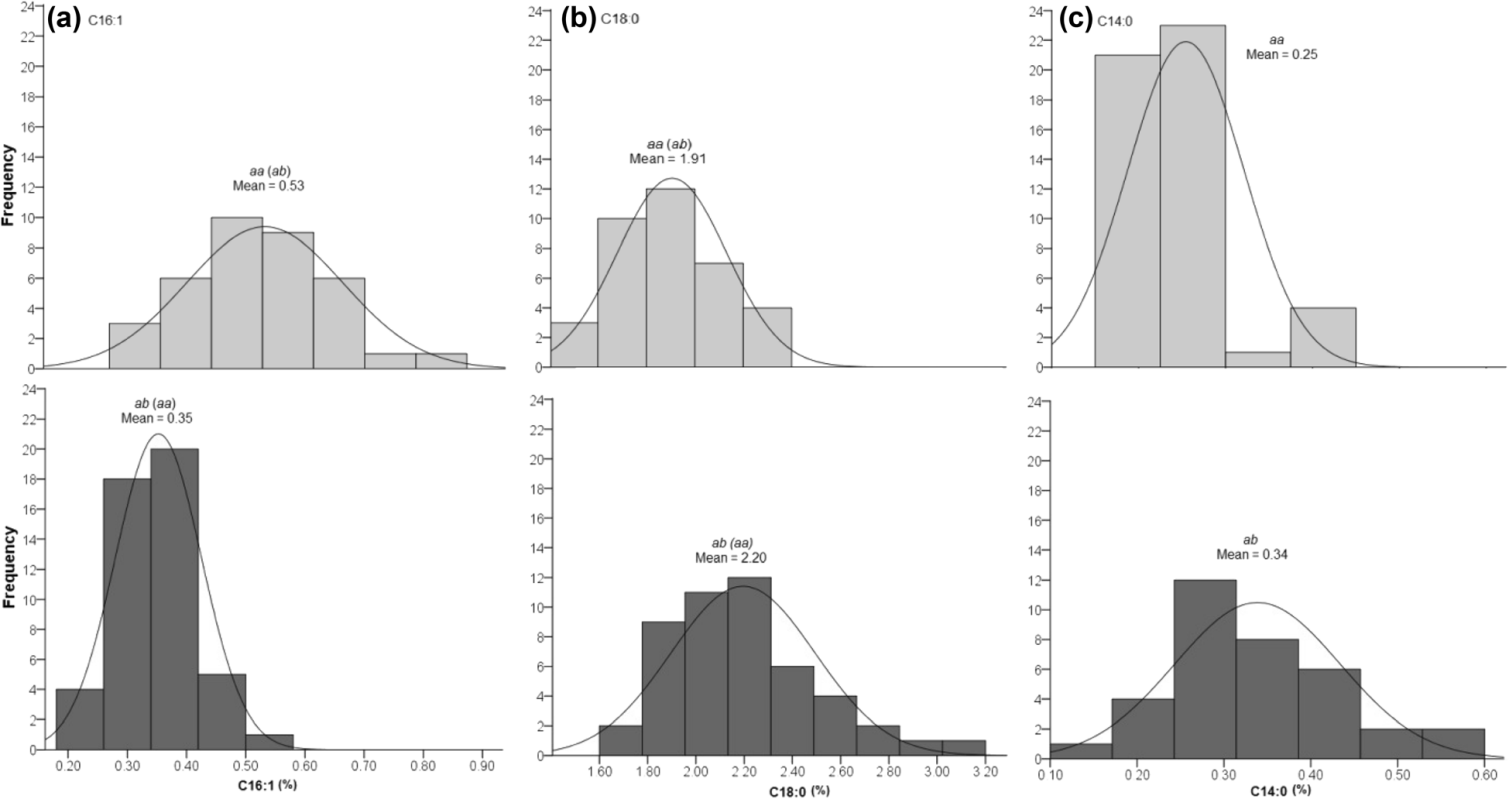
Distribution of phenotypes categorized based on the genotypes of the closest markers linked to the observed QTLs in LGT9. The upper panel (light grey) is of hybrids with genotype *ab* (*aa*) while the lower panel (dark grey) shows the phenotypes observed in genotype *aa* (*ab*) in SNPM00331 (SNPM00922) for C16:1 (**a**) and C18:0 (**b**) and, SNPM00343 for C14:0 (**c**)

For group 9 (Group 15 in [8]), three genome-wide significant QTLs for C14:0, C16:1 and C18:0 were identified and the interval was flanked by markers EAAG/MCAT-221 and P4T8I, as reported previously. The current map position for EAAG/MCAT-221 was determined using a less stringent mapping parameter (Fig. 2, b). The interval between EAAG/MCAT-221 and P4T8I contained three SNP markers, which reduced the gap to 2.8 cM/markers from the previous 5.8 cM. For C16:1 and C18:0, the QTL peak co-localized with markers SNPM00922 and SNPM00331 at map position 22.0 cM. Using GM, the marker effect was estimated to be 0.08 % (*p* = 0.0) and −0.03 % (*p* = 0.0000006) for C16:1 and C18:0, respectively. When the genotypes of the two markers were analysed, the *ab* and *aa* profiles showed significant differences (*p* < 0.05 *T*-test, SPSS 16.0) for C16:1 and C18:0 contents in the OxG hybrids (Fig. 3, a & b). For SNPM00922 (SNPM00331), the homozygous *aa* (*ab*) genotype showed an average 0.53 % (SD = 0.1) for C16:1 and 1.91 % (SD = 0.2) for C18:0 content which differed from the heterozygous *ab* (*aa*) genotypes (0.35 % ± 0.07 for C16:1 and 2.20 % ± 0.3 for C18:0). However, for C14:0, the closest marker was SNPM00343 (located at 17.2 cM) with a minor effect of −0.03 % (*p* = 0.0) and which also showed a significant difference in C14:0 content between the *aa* (0.25 % ± 0.07) and *ab* (0.34 % ± 0.1) genotypes (Fig. 3, c).

For LGOT4 (similar to Group 3 in [8]) QTL was detected for C18:0 in the 158.4–176.5 cM interval flanked by SNPM02160 and SNPM00121 (Fig. 2, c). The closest marker SNPM00121 at 165.1 cM, had a positive effect of 0.02 % (*p* = 0.0) on the trait, as estimated by GM. On LGT2 (Group 8 in the previous study), three markers (SNPM04410, SNPM01906 and SNPM04068) showed significant association with C14:0 (Fig. 2, d). These markers, co-localized with the QTL peak at map position 61.2 cM, were found at a distance 36.5 cM from the previously detected QTL (indicated by EACA/MCAA-270 and EAAC/MCAC-133). In addition, two putative QTLs for C18:2 were detected on LGs T3 (46.9–65.2 cM) and OT6 (38.9–54.5 cM) (Fig. 2, e & f). The putative (chromosome-wide) QTLs were considered in this case as the regions concerned also explained similar QTLs in other studies (described below). A summary of the QTL results is presented in Table 2.

**Table 2:** QTLs linked to iodine value (IV) and fatty acid composition (FAC) in the OxG interspecific mapping population. QTLs identified using Interval Mapping (IM), the Multiple-QTL Model (MQM), the Kruskal-Wallis non-parametric tests (KW) and G Model (GM)

Trait	IM					MQM		KW		GM	
	QTL interval (cM)	QTL peak (cM)	QTL peak (LOD)	Closest marker	Var (%)	LOD	Var (%)	K-value	*p*-value	Marker effect	*p*-value
IV (GW = LOD4.3)											
OT1^a^	102.3–151.5	149.6	17.4	SNPM04501	60.8	17.6	61.2	51.1	0.0001	2.16 (+)	0.0000000
				SNPM00967						2.16 (+)	0.0000000
				SNPM00144						2.16 (+)	0.0000000
				SNPM01034						2.16 (−)	0.0000000
				SNPM00150						2.16 (−)	0.0000000
				PA5_oSSR^a^						2.15 (−)	0.0000000
				PA3_oSSR^a^						2.18 (−)	0.0000000
				SNPE00431						2.17 (+)	0.0000000
				sPSc00328^a^						2.15 (+)	0.0000000
C14:0 (GW = LOD3.7)											
OT1^a^	140.7–151.5	149.6	6.2	SNPM04501	48.3	5.7	26.5	24.9	0.0001	0.04 (−)	0.0000000
				SNPM00967						0.04 (−)	0.0000000
				SNPM00144						0.04 (−)	0.0000000
				SNPM01034						0.04 (+)	0.0000000
				SNPM00150						0.04 (+)	0.0000000
				PA5_oSSR^a^						0.04 (+)	0.0000000
				PA3_oSSR^a^						0.04 (+)	0.0000000
				SNPE00431						0.04 (−)	0.0000000
				sPSc00328^a^						0.04 (−)	0.0000000
T2	61.2–62.1	61.2	4.1	SNPM04068	20.1	4.3	22.9	15.1	0.0005	0.03 (+)	0.0000000
				SNPM01906						0.03 (+)	0.0000000
				SNPM04410						0.03 (−)	0.0000000
T9	17.2–32.6	17.2	4.5	SNPM00343	21.9	4.0	21.5	17.7	0.0001	0.03 (−)	0.0000000
C16:0 (GW = LOD4.9)											
OT1^a^	102.3–151.5	149.6	21.5	SNPM04501	69.4	22.3	70.2	55.2	0.0001	2.41 (−)	0.0000000
				SNPM00967						2.41 (−)	0.0000000
				SNPM00144						2.42 (−)	0.0000000
				SNPM01034						2.41 (+)	0.0000000
				SNPM00150						2.41 (+)	0.0000000
				PA5_oSSR^a^						2.37 (+)	0.0000000
				PA3_oSSR^a^						2.35 (+)	0.0000000
				SNPE00431						2.42 (−)	0.0000000
				sPSc00328^a^						2.40 (−)	0.0000000
C16:1 (GW = LOD4.3)											
T9	2.9–32.6	22.0	10.7	SNPM00331	44.2	10.7	44.2	36.0	0.0001	0.08 (−)	0.0000000
				SNPM00922						0.08 (+)	0.0000000
C18:0 (GW = LOD3.8)											
OT1^a^	140.7–151.5	151.1	6.4	sPSc00314^a^	30.5	6.8	30.9	22.1	0.0001	0.14 (−)	0.0000001
OT4	158.4–176.5	165.1	4.9	SNPM00121	54.2	4.8	23.5	16.6	0.0001	0.02 (+)	0.0000020
T9	13.4–24.8	22.0	5.0	SNPM00331	23.8	5.0	23.8	19.0	0.0001	0.03 (+)	0.0000003
				SNPM00922						0.03 (−)	0.0000005
C18:1 (GW = LOD4.0)											
OT1^a^	112.6–151.5	149.6	16.3	SNPM04501	59.5	16.9	60.1	49.5	0.0001	2.29 (+)	0.0000000
				SNPM00967						2.17 (+)	0.0000000
				SNPM00144						2.18 (+)	0.0000000
				SNPM01034						2.17 (−)	0.0000000
				SNPM00150						2.29 (−)	0.0000000
				PA5_oSSR^a^						2.39 (−)	0.0000000
				PA3_oSSR^a^						2.06 (−)	0.0000000
				SNPE00431						2.07 (+)	0.0000000
				sPSc00328^a^						2.30 (+)	0.0000000
C18:2 (GW = LOD3.8)											
T3 (CW = LOD1.7)	46.9–65.2	63.3	2.1	SNPM00462	10.9	2.3	11.5	8.9	0.0050	0.17 (+)	0.0017218
OT6 (CW = LOD2.7)	38.9–54.5	45.8	3.1	SNPM05061	35.2	3.3	40.2	11.0	0.0010	0.16 (−)	0.0000052

^a^After fine mapping

GW: 95 % Genome-wide significant LOD threshold

CW: 95 % Chromosome-wide significant LOD threshold

### Fine-mapping of the QTLs on LGOT1

The map interval 106.4–136.2 cM (between pOP-CB00075a and EAGG/MCAT-198) on LGOT1, revealed multiple QTLs (IV, C14:0, C16:0, C18:0 and C18:1) with a relatively large proportion of phenotypic variation explained (30.5–69.4 %) and was of particular interest. Admittedly, the phenotypic variation could have been overestimated due to the limited size of the mapping family, leading to a Beavis effect [32, 33]. The markers in this region were mapped to the P5 genome build [18]. To improve the efficiency of finding the QTL corresponding region in the genome build, additional markers that fit into the region were also obtained from an independent *E. guineensis* map (LGDP1) [11]. This resulted in 29 markers (including 13 SNPs from LGDP1) mapping to scaffolds p5_sc00001 and p5_sc00104 with high identities (95–100 %) and e-values 0 – 1e-167. The total physical coverage of the QTL interval on p5_sc00001 (total 22,100,610 bp) and p5_sc00104 (total 2,594,271 bp) were 6,127,438 bp (27.7 %) and 1,689,644 bp (65.1 %), respectively.

**Fig. 4:**
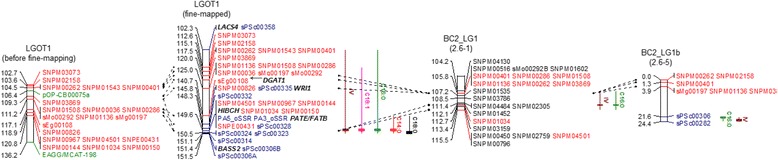
Fine-mapping of QTL intervals with candidate markers and cross-validation of QTLs in two independent BC_2_ crosses. Similar QTLs between LGOT1 (OxG mapping population) and LGs 1 and 1b of BC_2_ validation crosses (2.6–1 and 2.6–5) are indicated by common markers (in red) and candidate SSR markers (in blue)

The BLAST results for the QTL corresponding region identified significant similarity to one TF and a number of genes associated with the FA and TAG biosynthesis pathways [14, 15]. This included diacylglycerol acyltransferase (*DGAT1*) and long chain acyl-CoA synthetase (*LACS4*) in p5_sc00001. In p5_sc00104, sodium/metabolite cotransporter (*BASS2*), palmitoyl-ACP thioesterase (*PATE/FATB*), 3-hydroxyisobutyryl-CoA hydrolase-like protein 3 (*HIBCH*) and AP2-like ethylene-responsive TF (*WRI1*) were found. For fine mapping the QTL confidence interval, SSRs (with nomenclature sPSc) were mined and developed from the candidate gene introns (12 SSRs) and regions (11 SSRs) flanked at 1,096–25,202 bp before the 5’ start codon (upstream) and 351–4,095 bp downstream (after the 3’ stop codon) of the genes. In addition, six SSRs were developed specifically to fill in the gaps in the identified interval and therefore were not located near to any candidate gene. Of the 29 developed candidate SSRs, 12 were polymorphic and mapped back to the QTL interval on LGOT1. Unfortunately, five SSRs for *DGAT1* were not polymorphic and could not be mapped onto LGOT1. Refined QTL intervals for IV, C14:0, C16:0, C18:0 and C18:1 as well as the map positions of the candidate genes and TF are illustrated in Fig. 4.

**Fig. 5:**
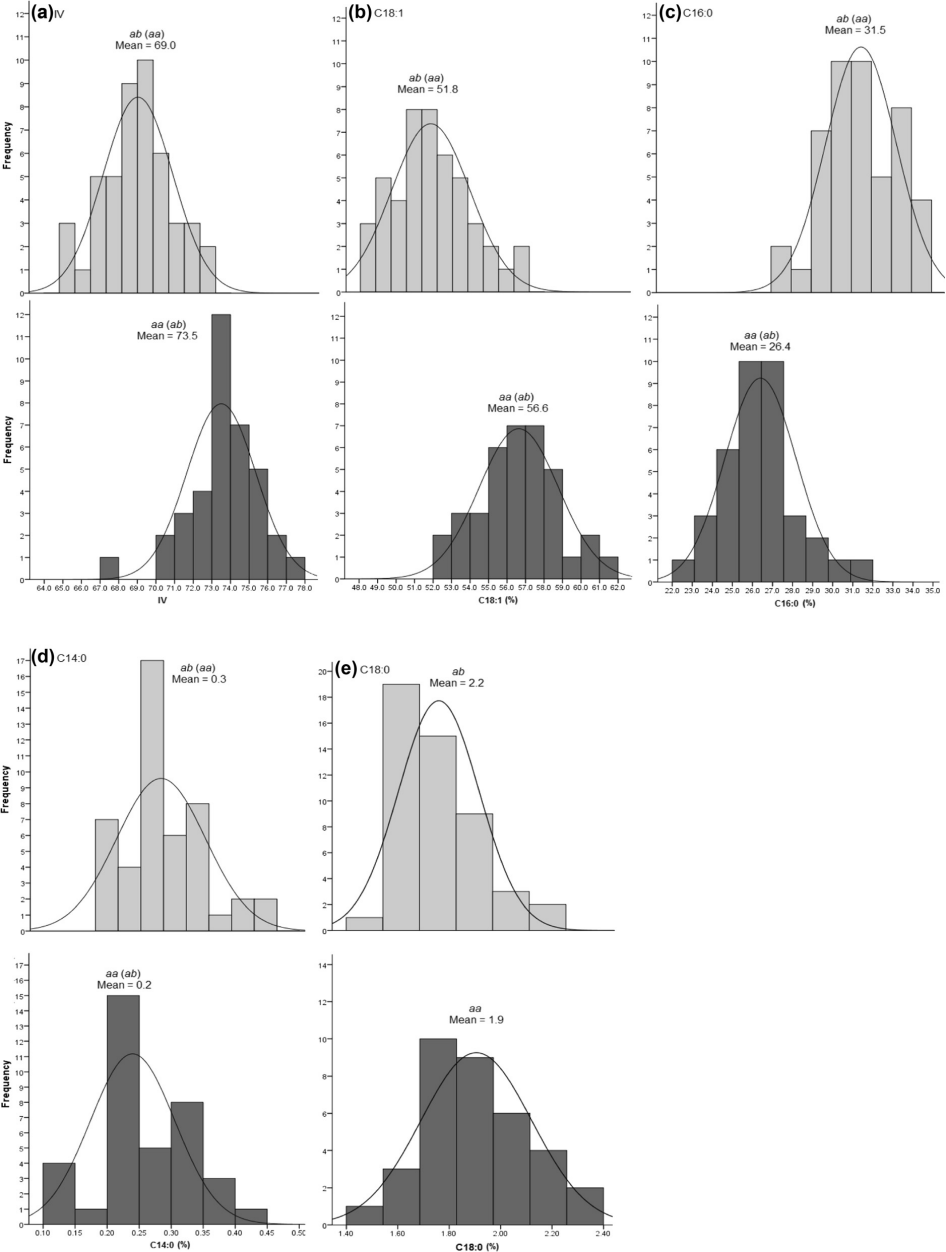
Distribution of IV and FAC phenotypes in OxG hybrids genotyped by the closest markers linked to the QTLs on LGOT1. The upper panel (light grey) is of hybrids with genotype *ab* (*aa*) while the lower panel (dark grey) shows the phenotypic distribution of genotype *aa* (*ab*) in SNPM04501, SNPM00967, sPSc00328, SNPM00144 or SNPE00431 (SNPM01034, SNPM00150, PA5_oSSR or PA3_oSSR) for IV (**a**), C18:1 (**b**), C16:0 (**c**) and C14:0 (**d**). For C18:0 (**e**), the *ab* and *aa* genotypes are observed in sPSc00314

After fine-mapping with candidate markers, slight increases in the LOD scores were observed for markers in the QTL intervals. For IV, significant QTL with LODs 6.3–17.4 were revealed in the 113–161.8 cM interval on LGOT1. Two candidate genes, *HIBCH* and *PATE/FATB*, together with three candidate SSRs (PA5_oSSR, PA3_oSSR and sPSc00328) and six existing SNPs (SNPM04501, SNPM00967, SNPM00144, SNPM01034, SNPM00150 and SNPE00431) co-localized directly with the QTL peak (at 149.6 cM). No recombination event was observed between the nine markers, possibly due to the limited size of the OxG population and the short distance (~975 kbp) on the physical map. Using GM, the nine markers were detected to have a significant effect close to 2.20 (*p* = 0.0) (Table 2). This result indicated that the maternal UP1026 genotype (*aa*) had an increasing effect on IV compared to the T128 genotype (*ab*) for SNPM04501, SNPM00967, sPSc00328, SNPM00144 or SNPE00431. In contrast, *aa* showed reduced levels of IV compared with *ab* when genotyped with SNPM01034, SNPM00150, PA5_oSSR or PA3_oSSR (Fig. 5, a). The difference in mean IV between the two genotypes improved to 4.47 (73.5 ± 1.9 *vs.* 69.0 ± 1.9) compared to 3.67 estimated previously using the RFLP marker, pOP-CB00075a by Singh et al. [8].

The same group of markers were also closely linked to the QTL for C18:1, with similar allelic effects as observed on IV (Fig. 5, b). These markers detected a greater difference (4.8 %) in C18:1 content compared to the 2.7 % explained by pOP-CB00075a previously. Another QTL revealed by the same group of markers was for C16:0 content. The estimated marker effect of 2.4 % (*p* = 0.0) had increasing alleles from the other parent, compared to that for IV and C18:1 which agrees with the strong negative correlation between the levels of saturated (C16:0) and unsaturated FAs (IV and C18:1). The difference in the C16:0 mean values of the homozygous and heterozygous genotypes also increased to 5.1 % from the 3.8 % previously reported (pOP-CB00075a). In addition to the three major QTLs, these markers also had a minor effect of 0.04 % (*p* = 0.0) on C14:0 with the increasing alleles inherited in the same direction as C16:0. The difference in C14:0 content between genotypes *aa* (0.24 % ± 0.07) and *ab* (0.33 % ± 0.09) was also relatively low at 0.1 %. With respect to C18:0, the refined QTL interval (140.7–151.5 cM) revealed a new candidate SSR marker sPSc00314 at the QTL peak with a negative effect of 0.14 % (p = 0.0000001). A difference of 0.3 % in C18:0 was observed between the *aa* and *ab* genotypes of sPSc00314 (Fig. 5, e). On the genome scaffold (p5_sc00104), sPSc00314 was located between *PATE/FATB* and *BASS2* with an estimated distance of ~ 247 kbp and 68 kbp, respectively. Therefore, it is still unclear at this point which of the genes, if any, is influencing the minor QTL for C18:0.

### Validation of QTLs for IV and C16:0 in BC_2_

In this study, the QTLs detected for IV and C16:0 in the OxG mapping population were tested for validation in the two BC_2_ crosses−2.6–1 and 2.6–5. In 2.6–1, a major QTL was detected for IV with LOD8.7 on LG1. The 11.3 cM confidence interval (104.2–115.5 cM) containing one SSR and 20 SNPs was then aligned to LGOT1 (in OxG) using nine transferable markers (SNPM00286, SNPM00401, SNPM01508, SNPM01136, SNPM00262, SNPM03869, SNPM01034, SNPM03159 and SNPM04501) which clearly showed that the same region was affecting IV in both crosses (Fig. 4). Of these markers, SNPM01034 underlying the QTL peak revealed three genotypes – *aa*, *ab* and *bb* – in the 2.6-1 palms. The *aa* genotype, similar to that observed in OxG, differentiated the IV mean (67.3 ± 2.0) from the *bb* genotype (64.1 ± 1.9) with *p* = 0.011 but, no significant difference observed in genotypes *aa vs. ab* (*p* = 0.228) and *bb vs. ab* (*p* = 0.087). The same marker SNPM01034 (LOD4.3) was also detected flanking a putative QTL peak for C16:0 with minor effect.

The QTLs with overlapping confidence intervals for IV and C16:0 were also identified in the 2.6–5 cross on a subgroup of LG1 (labelled as LG1b). When compared with LGOT1 in OxG, the same QTL region encompassing the common marker sPSc00306 was also located at the end of the LG1b. For IV, the QTL peak showed a high LOD score (5.6) with the confidence interval (21.6–24.4 cM) and contained two candidate SSR markers, sPSc00306 and sPSc00282, with estimated effects of 1.5–1.7 IV units. For the closest marker sPSc00306, the maternal genotype (*aa*) showed significantly (*p* = 0.05) lower unsaturation (IV) – 62.4 (±2.3) compared to 65.6 (±3.5) in the paternal genotype (*ab*). The sPSc00306 also showed a significant effect of 0.9 % (*p* = 0.0021634) and the *aa* and *ab* genotypes revealed a significant (*p* = 0.05) mean difference (36.0 % ± 2.1 *vs*. 33.6 % ± 3.2) in C16:0 content*.*

### Additional QTLs detected in the BC_2_

The 2.6–1 and 2.6–5 crosses revealed additional QTLs for C18:2 on LG4 which were not detected in OxG. For 2.6–5, the QTL in the confidence interval 3.9–9.4 cM enclosed seven markers (SNPM00203, SNPM00151, SNPM00249, SNPM00348, SNPM02910, SNPM01114 and SNPM04449). Of these, the closest flanking markers to the QTL peak (at 9.4 cM) were SNPM04449 and SNPM01114 with estimated effects of 0.9 % (*p* = 0.0008687) and 0.5 % (*p* = 0.0018213), respectively. In the 2.6–1 cross, similar QTL (indicated by common markers) although significant at the chromosome-wide level rather than the genome-wide level were also detected on LG4 with LODs ranging from 3.4–3.8. The left-right flanking markers were SNPM00971, SNPM04449, SNPM00203, SNPM00348 and SNPM00249 at 3.4 cM and SNPM00563, SNPM00692 and SNPM00151 at 5.5 cM (Additional file 5).

In comparison, more new QTLs were detected in 2.6–1 than in 2.6–5. These include i. C18:1 (22.7–24.6 cM on LG4), ii. C18:1 (35.6–38.7 cM on LG8), iii. IV (63.1–68 cM on LG15), iv. C16:1 (59.5–66.6 cM on LG15) and, v. C18:2 (53.2–60.3 cM on LG15). All were minor QTLs, significant at the 95 % chromosome-wide level.

## Discussion

This study extended the mapping of QTLs linked to IV and FAC in the OxG interspecific mapping population initiated by Singh et al. [8]. The preliminary map constructed with 252 markers localized 12 QTLs mostly loosely flanked by AFLP and RFLP markers. Therefore, in this study, a 4.7x more saturated genetic map was used to localize the QTLs and improve coverage. A total of 10 genome-wide and two putative QTLs for IV and various FAs were identified on six improved LGs (OT1, T2, T3, OT4, OT6 and T9), which included 11 of the QTLs detected previously. The results indicated that the current SSR and SNP-based genetic map was effective in revealing QTLs with the only exception being the minor QTL for C14:0 reported earlier on group 8 (the present LGT2). However, a new putative QTL was revealed for C14:0 at the nearby region of the same LG. The shift in location could indicate a more precise estimation of the QTL with the improved map resolution on LGT2, with a similar situation having been reported in eggplant [34].

The detected QTLs at this stage can only be considered to be specific to the OxG cross which would limit application of the markers for MAS. Mapping families created via a sequential series of backcrosses and self-pollination would ideally help validate the QTLs as reported in rapeseed [35], maize [36, 37] and soybean [38]. However such mapping families are not readily available for a perennial crop like oil palm and it would take about 10 years to create each generation, with initial phenotyping [39, 40], apart from the huge investment required in land, labour and management. It is for this reason that, to date, QTLs for IV and FAC in oil palm have only been reported in single crosses involving a BC_1_, *tenera* x *dura* F_1_ [16, 17] and an OxG hybrid [8]. In this study, we used two independent BC_2_ pseudo-crosses (2.6–1 and 2.6–5) created by introgression of *E. oleifera* into T128 germplasm materials to try to validate the QTLs detected in the OxG hybrid population. To our knowledge, this is the first attempt to validate QTLs for FAC in oil palm of similar genetic backgrounds.

The two BC_2_ crosses are related by pedigree and also genetically linked with the T128 germplasm (Fig. 1). Here we adopted a strategy to evaluate the QTLs through an independent analysis with subsequent comparison of their locations. However, our results showed that only the major QTLs for IV and C16:0 were successfully cross-validated through a common location, and all similarly revealed a large proportion of phenotypic variance (43.0–53.0 % for IV and 31.0–62.0 % for C16:0) in both 2.6–1 and 2.6–5. The common QTLs in BC_2_ revealed that the different genotypes of closely linked markers also showed distinct levels for IV and C16:0 content. The finding is of interest as IV represents the cumulative unsaturation in palm oil and it has always been used as an indicator in selecting for higher unsaturation overall in breeding programs [41], whereas the QTL for C16:0 can complement the prediction by indicating the palmitic (saturation) level. It is however noted that the QTL for C18:1 could not be cross-validated in the two BC_2_ populations even though C18:1 is a major component contributing to IV and in this study was highly correlated with IV and negatively correlated with C16:0. The small size of the mapping families could have limited the detection power for the QTL analysis, resulting in the lack of detection of the QTLs in the BC_2_ crosses. A similar observation was made in maize about the effect of population size when a sub sample (about 1/3 of the larger population) only revealed about 1/5 of the QTLs observed in larger samples [42].

In addition to validating QTLs in the interspecific BC_2_, eight of the identified QTLs were similar to those reported previously using either a BC_1_ or a *tenera* × *dura* cross [16, 17]. Multiple QTLs for C14:0, C16:0, C18:0 and C18:1 were reported previously in the respective intervals 0–25.6, 0–7.0, 0–5.8 and 0 – 6.0 cM on LG9 in the *tenera* x *dura* mapping family. The intervals were aligned with the current QTLs on LGT9 using three common markers, mEgCIR3787, mEgCIR3592 and mEgCIR3305, pointing to a similar map region. In the previous studies by Montoya et al. [16, 17], the intervals in LG4 (199.8–234.4 cM and 201.8–216.0 cM) were reported to be linked to C18:0 and C18:1, respectively. Similar regions affecting the QTLs were also detected in the present OxG (C18:0) and 2.6–1 (C18:1) crosses, determined using the common markers mEgCIR3535, mEgCIR1753 and mEgCIR3310 mapped in both the studies. Another possible common QTL (determined using markers mEgCIR3649, mEgCIR3282 and mEgCIR0800) was C14:0 on LGT2 which was also previously reported on the same LG in the BC_1_ [16]. The putative QTL linked to C18:2 on LGT3 and LGOT6 were similar to that reported previously. The QTL on LGT3 matched the genomic region reported previously for a BC_1_ population [17], as revealed by the common marker mEgCIR0425, mapping in the same region. With respect to LGOT6, the QTL region was very close to that reported previously for the same trait by Singh et al. [8]. The above comparison has provided useful information for comparing QTLs linked to FAC by different research groups. We compiled and compared a total 60 QTLs linked to IV and FAC (14 in OxG, 12 in two BC_2_, 15 in *tenera* x *dura* and 19 in BC_1_) across the different mapping families. However, very low percentages of the QTLs were common across two (20.0 %) to three (2.0 %) of the crosses and none at all in more than three crosses. This highlights that in QTL analysis, even for a highly heritable trait such as FAC, favourable alleles are likely to be population-specific requiring careful selection for implementation in MAS. Nevertheless, this study revealed a core set of markers (SNPM04501, SNPM00967, sPSc00328, SNPM00144, SNPE00431, SNPM01034, SNPM00150, PA5_oSSR and PA3_oSSR) that can be useful for MAS, especially in genetic backgrounds involving the T128 and *E. oleifera* parental palms. As the palm materials are commonly available and accessible to the oil palm industry, the markers will have practical application for selecting favourable alleles for unsaturation (*vs*. saturation) in interspecific hybrids and their backcrosses.

Detection of similar QTLs for IV and C16:0 in both OxG and BC_2_ allowed the underlying QTL interval to be compared to the recently published oil palm genome build [18]. This study is also the first to describe attempts at fine-mapping of targeted QTL regions for IV and various FAC, by exploiting the whole genome sequence data of oil palm. The genomic region of about 6 kbp showed the presence of an interesting gene with high similarity to *PATE/FATB* in oil palm [GenBank: XM_010916712.1, XM_010916714.1, DQ422858, AF424808, AF430248, AF147879 and AF541880] and coconut [GenBank: JF338904 and JF338903]. Comparison of genetic maps using the common markers mEgCIR0008, mEgCIR3428 and mEgCIR3819 estimated that the current map position of *PATE/FATB* in LGOT1 is in accordance with Montoya et al. [16]. However, the authors did not associate the gene or region around the gene with any QTL for IV or FAC.

In oil palm, *PATE/FATB* encodes the palmitoyl-ACP thioesterase that actively releases C16:0 from C16:0-ACP and allows the resulting non-esterified FA to be incorporated into TAG [15, 43] and is one of the main factors responsible for the relatively high saturation levels in palm oil [44]. A similar role was reported for *PATE/FATB* in oil biosynthesis in cotton seed [45]. Apart from a strong substrate preference for C16:0, the substrate specificity of palmitoyl-ACP thioesterase also extends to other saturated acyl-ACPs, including C14:0- and C18:0-ACPs [46, 47]. This was shown in other species by over-expression of *PATE/FATB* in *Arabidopsis thaliana* [48, 49] and canola seeds [50] which also had an elevated concentration of C14:0. This is in agreement with the strong positive correlation observed in this study between the C16:0 and C14:0 phenotypes. In contrast, the phenotypic data for levels of C16:0 had a strong negative correlation with C18:1 levels which indicates that a reduction of C16:0 is associated with increased C18:1. This was also observed in maize seeds where a mutated *FATB* allele caused a dramatic reduction of C16:0 in the oil and increased the level of unsaturated FAs [47]. The truncated *FATB* was reported responsible for the non-functioning of a critical catalytic domain and this could have resulted in the channelling of more C16:0-ACP for elongation and subsequent desaturation into unsaturated acyl chains, such as C18:1-ACP. It was also suggested that at least one of the palmitoyl-ACP thioesterase isoforms in oil palm can hydrolyze C18:1 efficiently [51]. This could also explain the co-localization of QTLs for IV, C14:0, C16:0, C18:0 and C18:1 on LGOT1 found in this study. Therefore, the next step is to evaluate the identified *PATE/FATB* gene in palms with varying contents of C16:0 and C18:1.

Another interesting region was at ~ 294 kbp (including non-sequenced gaps) from *PATE/FATB* (661,598 – 666,373 bp in p5_sc00104). This genomic region (~21 kbp) contained a gene with significant similarity to *HIBCH* in both oil palm [GenBank: XR_831088.1, XM_010916727.1, XM_010916728.1, XM_010916729.1 and XM_010916731.1] and date palm [GenBank: XM_008795208.1]. In oil palm, information about the role of *HIBCH* (found in mitochondrial) associated with FA and TAG biosynthesis is very limited. This enzyme is reportedly involved in the degradation of FAs (beta oxidation) in *Camelina sativa* [52] which takes place in mitochondrial and the peroxisomes. In coconut, *HIBCH* was reported as being down-regulated during the production and accumulation of oil [53].

Several other interesting genes and TF encoding key enzymes involved in both the FA and TAG pathways, including *BASS2*, *LACS4*, *DGAT1* and *WRI1*, were also found in proximity to the QTL peak. *BASS2* [GenBank: XM_010916675.1 and XM_010916676.1] is reported to play a major role in channelling pyruvate into plastids [54] for conversion into acetyl-CoA and malonyl-CoA – the main substrates to initiate synthesis of FA. At the final stage of FA biosynthesis, non-esterified FAs are converted into acyl-CoA by long chain acyl-CoA synthetase and later transferred through the plastid envelope into the ER for synthesis of TAGs [13, 55, 56]. On the current LGOT1, the long chain acyl-CoA synthetase family member 4 (*LACS4*) [GenBank: XM_010917352.1, XM_010927422.1, XR_832599.1 and XM_008790523.1] was identified at 112.9 cM close to the end of the QTL interval. In *A. thaliana*, *LACS4* showed greatest preference for the C16:0 substrate when expressed in *E. coli.* Substrate preference for various FAs was reported in the following order: C16:0 > C16:1 > C18:2 > C18:1 > C20:1 > C18:0 [56]. This may again explain the co-localization of QTLs for C16:0 with other FAs on LGOT1 where genes, such as *PATE/FATB* and *LACS4*, show pleiotropic effects contributing to the complex interactions with the substrates in FA synthesis.

In this study, *DGAT* is the only gene detected in the QTL region that is involved in formation of TAGs in the acyl-CoA-dependent (Kennedy) pathway [13, 57]. The ~ 28 kbp genomic region (between SNPM01136, sMg00197, SNPM01508, SNPM00286, sMo00292, SNPM00036 and sEg00108) has high sequence similarities to *DGAT1* in oil [GenBank: XM_010927169.1, XM_010927170.1, XM_010927171.1 and XM_010927172.1] and date palms [GenBank: XM_008794980.1 and XM_008794981.1]. In oil palm, *DGAT1* is known to interact with other acyltransferases, in particular, *DGAT2*, to synthesize TAG [14, 50]. It is generally agreed that *DGAT1* plays an important role in one of the oil palm lipid production pathways. However, information on the specificity and selectivity of each paralogue of *DGAT* in oil palm is still lacking and requires further study to establish their function and effect on the composition of FA in palm oil.

In addition to the key enzymes mentioned above, we identified a *WRI1* TF co-localized with two markers (sPSc00335 and SNPM00826) at 145.8 cM in the QTL region on LGOT1. The oil palm *WRI1* [GenBank: XM_010916833.1] has high similarity to that in date palm [GenBank: XM_008777490.1 and XM_008811881.1], plum [GenBank: XM_008223524.1], Euphrates poplar [GenBank: XM_011006767.1] and grape [GenBank: XM_010662724.1]. This TF has long been considered a master regulator directly influencing a number of enzymes in the FA and TAG synthesis pathways in plants [13, 57–61], including oil palm [14, 15].

## Conclusions

This study for the first time extensively compared the QTLs linked to IV and FAC across various interspecific genetic backgrounds. The detected QTLs, to some extent, are population specific although the major QTLs can be observed across related genetic backgrounds. A core set of markers with practical application in selection for higher unsaturation in FA levels were described. The candidate markers linked to the QTLs revealed significant allelic and genotypic differences associated with IV and FAC, suggesting that the markers are potentially useful in MAS, at least in the genetic backgrounds described. The fine-mapping approach employed in this study also proved effective in identifying candidate genes and a transcription factor affecting IV and FAC in palm oil. The identified genes and transcription factor, *HIBCH*, *PATE/FATB*, *BASS2*, *LACS4*, *DGAT1* and *WRI1* underlying the overlapping QTL confidence intervals for IV, C14:0, C16:0, C18:0 and C18:1, have provided valuable information on several potential candidate genes and a transcription factor which are known in other species to strongly influencing the biosynthesis of FA and TAG. Thus, to fully understand the interaction and effects of these genes, the listed candidate genes will be further studied to determine their expression levels in palms with different IV and FAC.

## Availability of supporting data

The sequence information for the SNP and SSR markers is available at http://genomsawit.mpob.gov.my

## Additional files

Additional file 1: Mapping candidate markers for various fatty acid related genes in the OxG linkage map. Candidate SNP markers (SNPE, in pink) for palmitoyl-ACP thioesterase (*PATE/FATB*), oleoyl-CoA desaturase (*FAD2*), linoleoyl-CoA desaturase (*FAD3*), enoyl-ACP reductase (*ENR1*) and stearoyl-ACP desaturase (*SAD*) mapped onto LGs OT1, T2, OT11, OT12 and T14. Candidate SSR markers (_oSSR and sPSc, in blue) also developed for ketoacyl-ACP synthase I (*KASI*), acetoacetyl-CoA thiolase (*AACT*), long chain acyl-CoA synthetase (*LACS4*), AP2-like ethylene-responsive TF (*WRI1*), 3-hydroxyisobutyryl-CoA hydrolase-like protein 3 (*HIBCH*), sodium/metabolite cotransporter (*BASS2*), *PATE/FATB* and *SAD*. (PDF 56 kb) Additional file 2: Pearson’s correlation coefficients for iodine value (IV) and fatty acid composition (FAC) in palm oil of OxG mapping population. (PDF 13 kb) Additional file 3: Pearson’s correlation coefficients for iodine value (IV) and fatty acid composition (FAC) in palm oil of BC_2_ (2.6-1) validation cross. (PDF 13 kb) Additional file 4: Pearson’s correlation coefficients for iodine value (IV) and fatty acid composition (FAC) in palm oil of BC_2_ (2.6-5) validation cross. (PDF 13 kb) Additional file 5: Co-linearity of markers between the OxG and BC_2_ (2.6-1 & 2.6-5) populations as observed in six linkage groups hosting QTLs (OT1, T2, T3, OT4, OT6 and T9). (PDF 96 kb)

## Abbreviations

BLASTN: similarity search of the NCBI nucleotide database using a nucleotide query
BLASTX: similarity search of the NCBI protein database using a translated nucleotide query
cM: centimorgan map distance
LOD: logarithm of odds
nt: NCBI nucleotide collection
nr: NCBI non-redundant protein sequences database
P5: *E. guineensis* (AVROS, *pisifera*) 5^th^ genome build
p5_scxxxxx: *E. guineensis* (AVROS, *pisifera*) 5^th^ genome scaffolds
PA*x*_oSSR: Candidate SSR marker for palmitoyl-ACP thioesterase
PCR: polymerase chain reaction
refseq_protein: NCBI reference proteins database
SD: standard deviation
SNPM*xxxxx*: single nucleotide polymorphism marker
SNPE*xxxxx*: candidate single nucleotide polymorphism marker
sPSc*xxxxx*: candidate simple sequence repeat marker
