# Supplementary material for: Fine-mapping and cross-validation of QTLs linked to fatty acid composition in multiple independent interspecific crosses of oil palm
Source: BMC Genomics. 2016 Apr 14;17:289. doi: 10.1186/s12864-016-2607-4 (PMC4832457; doi:10.1186/s12864-016-2607-4)
Supplement: Additional file 1: — Mapping candidate markers for various fatty acid related genes in the OxG linkage map. Candidate SNP markers (SNPE, in pink) for palmitoyl-ACP thioesterase (PATE/FATB), oleoyl-CoA desaturase (FAD2), linoleoyl-CoA desaturase (FAD3), enoyl-ACP reductase (ENR1) and stearoyl-ACP desaturase (SAD) mapped onto LGs OT1, T2, OT11, OT12 and T14. Candidate SSR markers (_oSSR and sPSc, in blue) also developed for ketoacyl-ACP synthase I (KASI), acetoacetyl-CoA thiolase (AACT), long chain acyl-CoA synthetase (LACS4), AP2-like ethylene-responsive TF (WRI1), 3-hydroxyisobutyryl-CoA hydrolase-like protein 3 (HIBCH), sodium/metabolite cotransporter (BASS2), PATE/FATB and SAD. (PDF 56 kb) [file 12864_2016_2607_MOESM1_ESM.pdf]

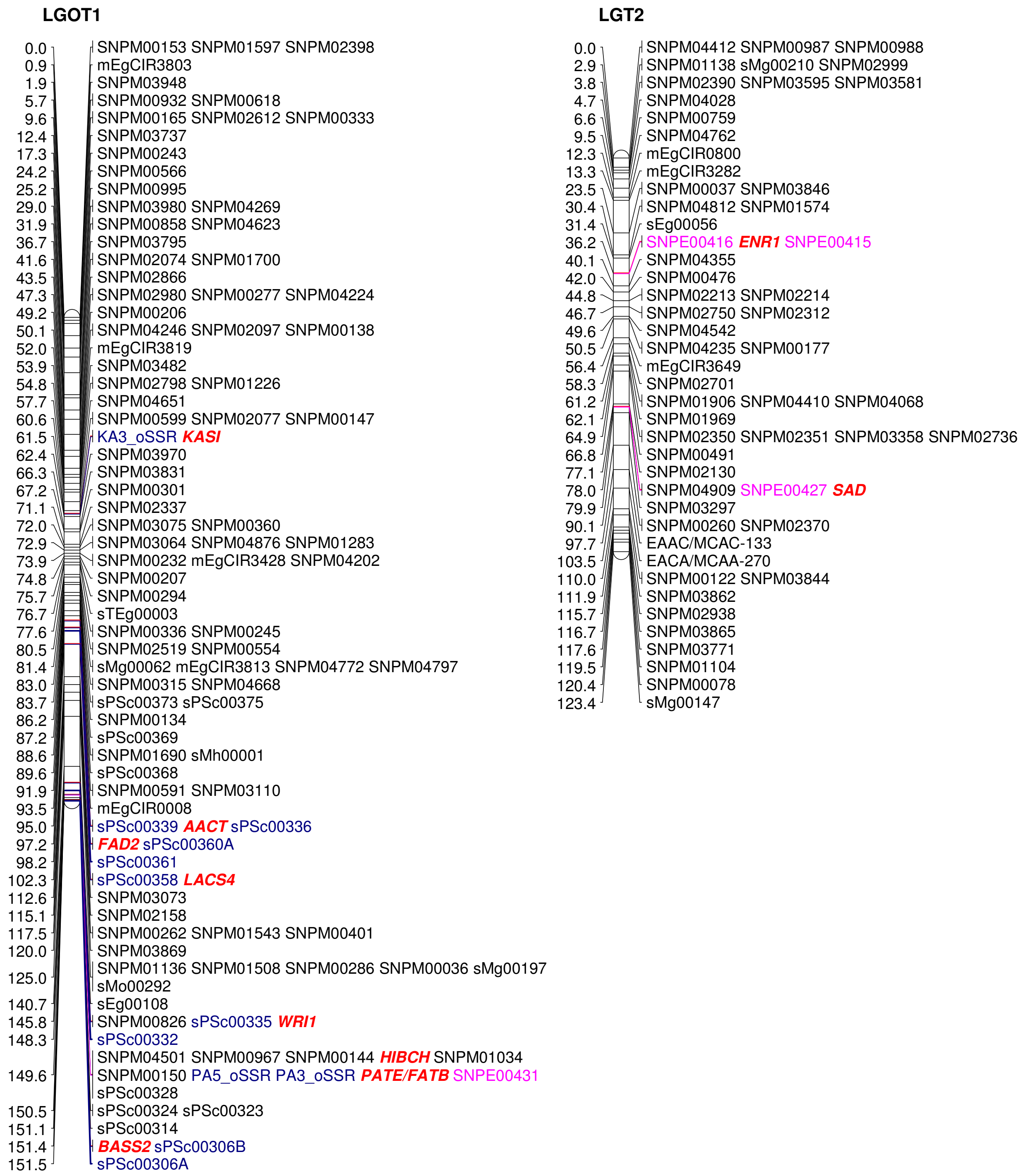

LGOT1
0.0 SNPM00153 SNPM01597 SNPM02398
0.9 mEgCIR3803
1.9 SNPM03948
5.7 SNPM00932 SNPM00618
9.6 SNPM00165 SNPM02612 SNPM00333
12.4 SNPM03737
17.3 SNPM00243
24.2 SNPM00566
25.2 SNPM00995
29.0 SNPM03980 SNPM04269
31.9 SNPM00858 SNPM04623
36.7 SNPM03795
41.6 SNPM02074 SNPM01700
43.5 SNPM02866
47.3 SNPM02980 SNPM00277 SNPM04224
49.2 SNPM00206
50.1 SNPM04246 SNPM02097 SNPM00138
52.0 mEgCIR3819
53.9 SNPM03482
54.8 SNPM02798 SNPM01226
57.7 SNPM04651
60.6 SNPM00599 SNPM02077 SNPM00147
61.5 KA3_oSSR KASI
62.4 SNPM03970
66.3 SNPM03831
67.2 SNPM00301
71.1 SNPM02337
72.0 SNPM03075 SNPM00360
72.9 SNPM03064 SNPM04876 SNPM01283
73.9 SNPM00232 mEgCIR3428 SNPM04202
74.8 SNPM00207
75.7 SNPM00294
76.7 sTEg00003
77.6 SNPM00336 SNPM00245
80.5 SNPM02519 SNPM00554
81.4 sMg00062 mEgCIR3813 SNPM04772 SNPM04797
83.0 SNPM00315 SNPM04668
83.7 sPSc00373 sPSc00375
86.2 SNPM00134
87.2 sPSc00369
88.6 SNPM01690 sMh00001
89.6 sPSc00368
91.9 SNPM00591 SNPM03110
93.5 mEgCIR0008
95.0 sPSc00339 AACT sPSc00336
97.2 FAD2 sPSc00360A
98.2 sPSc00361
102.3 sPSc00358 LACS4
112.6 SNPM03073
115.1 SNPM02158
117.5 SNPM00262 SNPM01543 SNPM00401
120.0 SNPM03869
125.0 SNPM01136 SNPM01508 SNPM00286 SNPM00036 sMg00197
sMo00292
140.7 sEg00108
145.8 SNPM00826 sPSc00335 WRI1
148.3 sPSc00332
149.6 SNPM04501 SNPM00967 SNPM00144 HIBCH SNPM01034
SNPM00150 PA5_oSSR PA3_oSSR PATE/FATB SNPE00431
sPSc00328
150.5 sPSc00324 sPSc00323
151.1 sPSc00314
151.4 BASS2 sPSc00306B
151.5 sPSc00306A
LGT2
0.0 SNPM04412 SNPM00987 SNPM00988
2.9 SNPM01138 sMg00210 SNPM02999
3.8 SNPM02390 SNPM03595 SNPM03581
4.7 SNPM04028
6.6 SNPM00759
9.5 SNPM04762
12.3 mEgCIR0800
13.3 mEgCIR3282
23.5 SNPM00037 SNPM03846
30.4 SNPM04812 SNPM01574
31.4 sEg00056
36.2 SNPE00416 ENR1 SNPE00415
40.1 SNPM04355
42.0 SNPM00476
44.8 SNPM02213 SNPM02214
46.7 SNPM02750 SNPM02312
49.6 SNPM04542
50.5 SNPM04235 SNPM00177
56.4 mEgCIR3649
58.3 SNPM02701
61.2 SNPM01906 SNPM04410 SNPM04068
62.1 SNPM01969
64.9 SNPM02350 SNPM02351 SNPM03358 SNPM02736
66.8 SNPM00491
77.1 SNPM02130
78.0 SNPM04909 SNPE00427 SAD
79.9 SNPM03297
90.1 SNPM00260 SNPM02370
97.7 EAAC/MCAC-133
103.5 EACA/MCAA-270
110.0 SNPM00122 SNPM03844
111.9 SNPM03862
115.7 SNPM02938
116.7 SNPM03865
117.6 SNPM03771
119.5 SNPM01104
120.4 SNPM00078
123.4 sMg00147

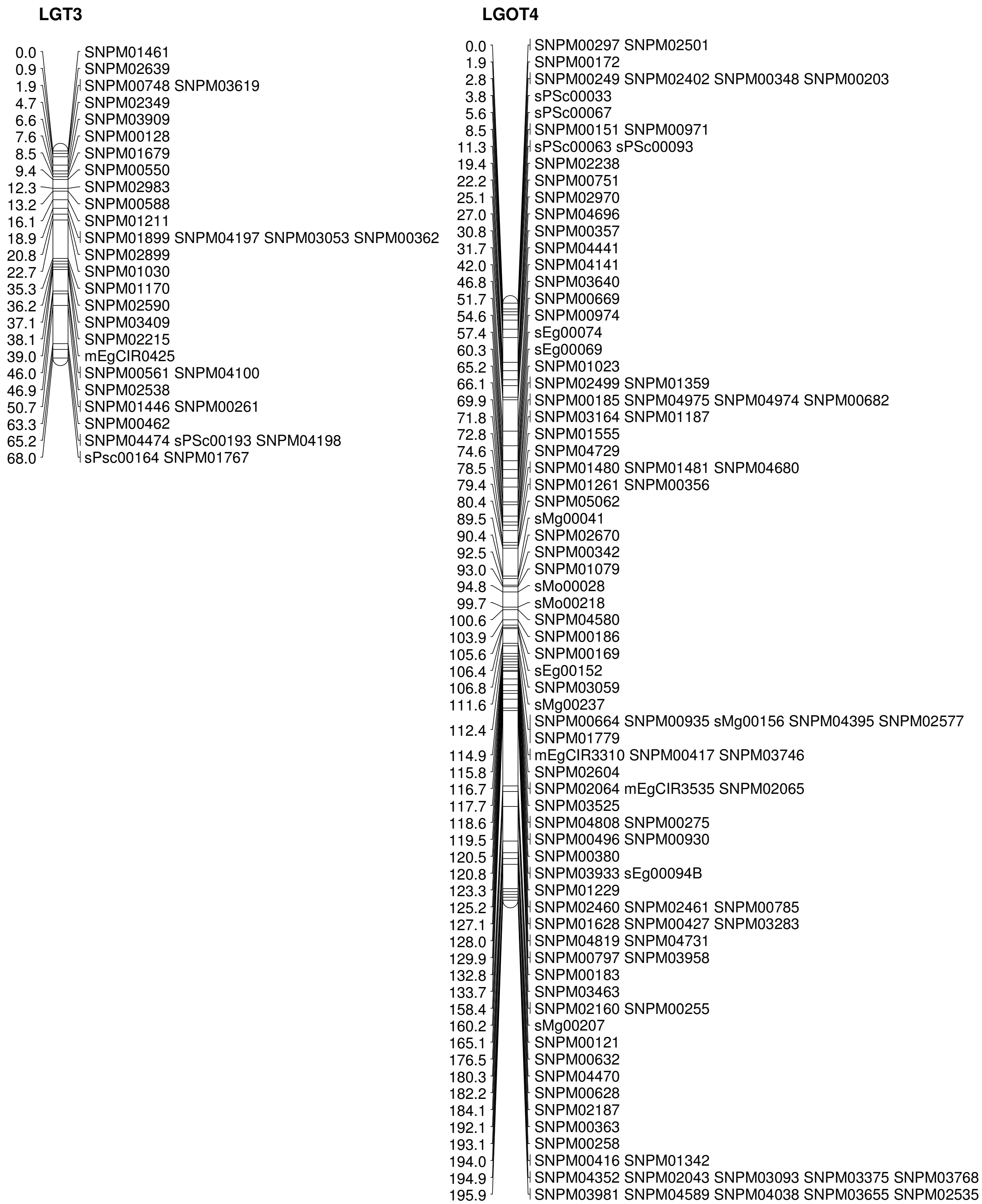
LGT3
0.0 SNPM01461
0.9 SNPM02639
1.9 SNPM00748 SNPM03619
4.7 SNPM02349
6.6 SNPM03909
7.6 SNPM00128
8.5 SNPM01679
9.4 SNPM00550
12.3 SNPM02983
13.2 SNPM00588
16.1 SNPM01211
18.9 SNPM01899 SNPM04197 SNPM03053 SNPM00362
20.8 SNPM02899
22.7 SNPM01030
35.3 SNPM01170
36.2 SNPM02590
37.1 SNPM03409
38.1 SNPM02215
39.0 mEgCIR0425
46.0 SNPM00561 SNPM04100
46.9 SNPM02538
50.7 SNPM01446 SNPM00261
63.3 SNPM00462
65.2 SNPM04474 sPSc00193 SNPM04198
68.0 sPsc00164 SNPM01767
LGOT4
0.0 SNPM00297 SNPM02501
1.9 SNPM00172
2.8 SNPM00249 SNPM02402 SNPM00348 SNPM00203
3.8 sPSc00033
5.6 sPSc00067
8.5 SNPM00151 SNPM00971
11.3 sPSc00063 sPSc00093
19.4 SNPM02238
22.2 SNPM00751
25.1 SNPM02970
27.0 SNPM04696
30.8 SNPM00357
31.7 SNPM04441
42.0 SNPM04141
46.8 SNPM03640
51.7 SNPM00669
54.6 SNPM00974
57.4 sEg00074
60.3 sEg00069
65.2 SNPM01023
66.1 SNPM02499 SNPM01359
69.9 SNPM00185 SNPM04975 SNPM04974 SNPM00682
71.8 SNPM03164 SNPM01187
72.8 SNPM01555
74.6 SNPM04729
78.5 SNPM01480 SNPM01481 SNPM04680
79.4 SNPM01261 SNPM00356
80.4 SNPM05062
89.5 sMg00041
90.4 SNPM02670
92.5 SNPM00342
93.0 SNPM01079
94.8 sMo00028
99.7 sMo00218
100.6 SNPM04580
103.9 SNPM00186
105.6 SNPM00169
106.4 sEg00152
106.8 SNPM03059
111.6 sMg00237
112.4 SNPM00664 SNPM00935 sMg00156 SNPM04395 SNPM02577
SNPM01779
114.9 mEgCIR3310 SNPM00417 SNPM03746
115.8 SNPM02604
116.7 SNPM02064 mEgCIR3535 SNPM02065
117.7 SNPM03525
118.6 SNPM04808 SNPM00275
119.5 SNPM00496 SNPM00930
120.5 SNPM00380
120.8 SNPM03933 sEg00094B
123.3 SNPM01229
125.2 SNPM02460 SNPM02461 SNPM00785
127.1 SNPM01628 SNPM00427 SNPM03283
128.0 SNPM04819 SNPM04731
129.9 SNPM00797 SNPM03958
132.8 SNPM00183
133.7 SNPM03463
158.4 SNPM02160 SNPM00255
160.2 sMg00207
165.1 SNPM00121
176.5 SNPM00632
180.3 SNPM04470
182.2 SNPM00628
184.1 SNPM02187
192.1 SNPM00363
193.1 SNPM00258
194.0 SNPM00416 SNPM01342
194.9 SNPM04352 SNPM02043 SNPM03093 SNPM03375 SNPM03768
195.9 SNPM03981 SNPM04589 SNPM04038 SNPM03655 SNPM02535

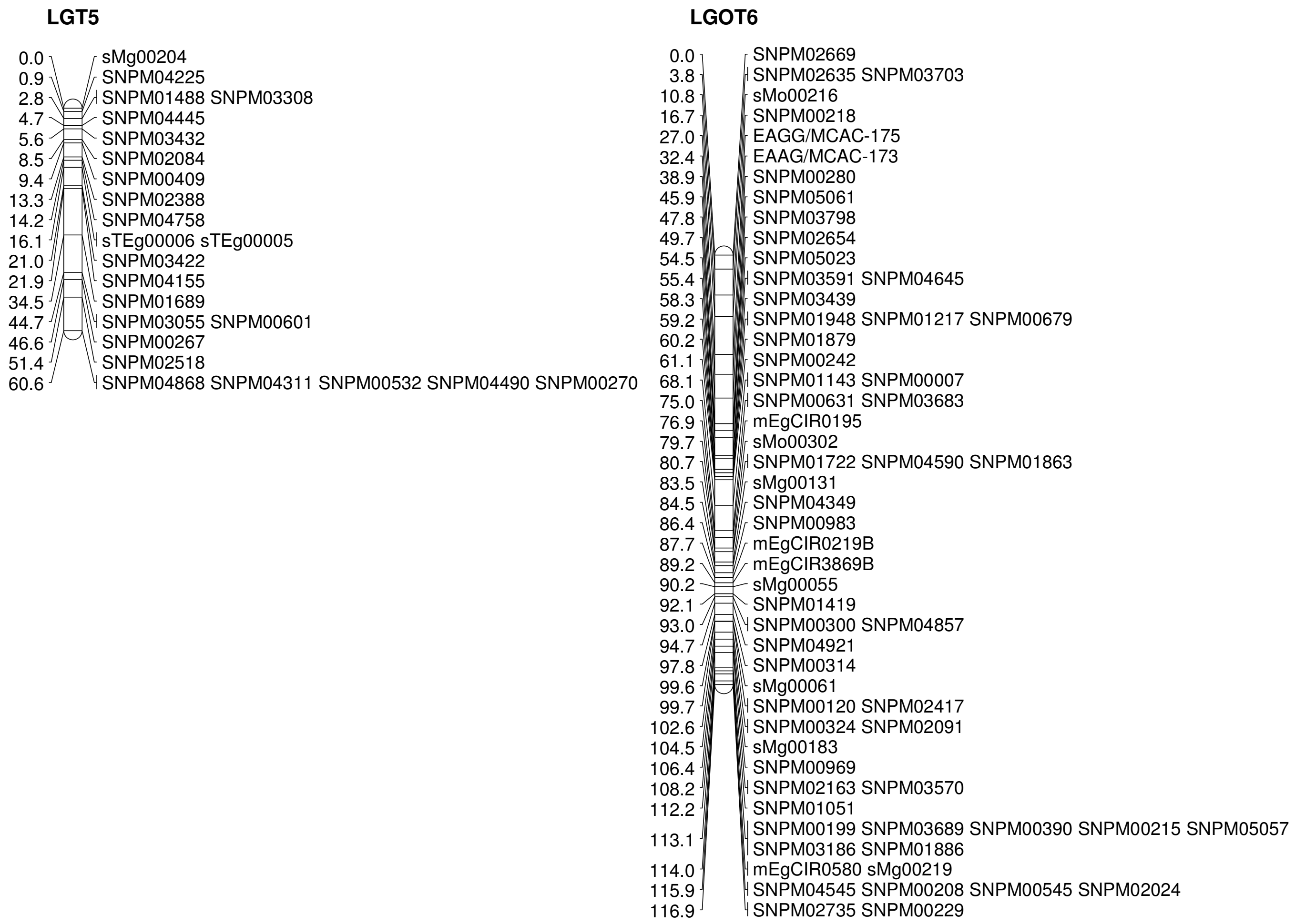
LGT5
0.0 sMg00204
0.9 SNPM04225
2.8 SNPM01488 SNPM03308
4.7 SNPM04445
5.6 SNPM03432
8.5 SNPM02084
9.4 SNPM00409
13.3 SNPM02388
14.2 SNPM04758
16.1 sTEg00006 sTEg00005
21.0 SNPM03422
21.9 SNPM04155
34.5 SNPM01689
44.7 SNPM03055 SNPM00601
46.6 SNPM00267
51.4 SNPM02518
60.6 SNPM04868 SNPM04311 SNPM00532 SNPM04490 SNPM00270
LGOT6
0.0 SNPM02669
3.8 SNPM02635 SNPM03703
10.8 sMo00216
16.7 SNPM00218
27.0 EAGG/MCAC-175
32.4 EAAG/MCAC-173
38.9 SNPM00280
45.9 SNPM05061
47.8 SNPM03798
49.7 SNPM02654
54.5 SNPM05023
55.4 SNPM03591 SNPM04645
58.3 SNPM03439
59.2 SNPM01948 SNPM01217 SNPM00679
60.2 SNPM01879
61.1 SNPM00242
68.1 SNPM01143 SNPM00007
75.0 SNPM00631 SNPM03683
76.9 mEgCIR0195
79.7 sMo00302
80.7 SNPM01722 SNPM04590 SNPM01863
83.5 sMg00131
84.5 SNPM04349
86.4 SNPM00983
87.7 mEgCIR0219B
89.2 mEgCIR3869B
90.2 sMg00055
92.1 SNPM01419
93.0 SNPM00300 SNPM04857
94.7 SNPM04921
97.8 SNPM00314
99.6 sMg00061
99.7 SNPM00120 SNPM02417
102.6 SNPM00324 SNPM02091
104.5 sMg00183
106.4 SNPM00969
108.2 SNPM02163 SNPM03570
112.2 SNPM01051
113.1 SNPM00199 SNPM03689 SNPM00390 SNPM00215 SNPM05057
SNPM03186 SNPM01886
114.0 mEgCIR0580 sMg00219
115.9 SNPM04545 SNPM00208 SNPM00545 SNPM02024
116.9 SNPM02735 SNPM00229

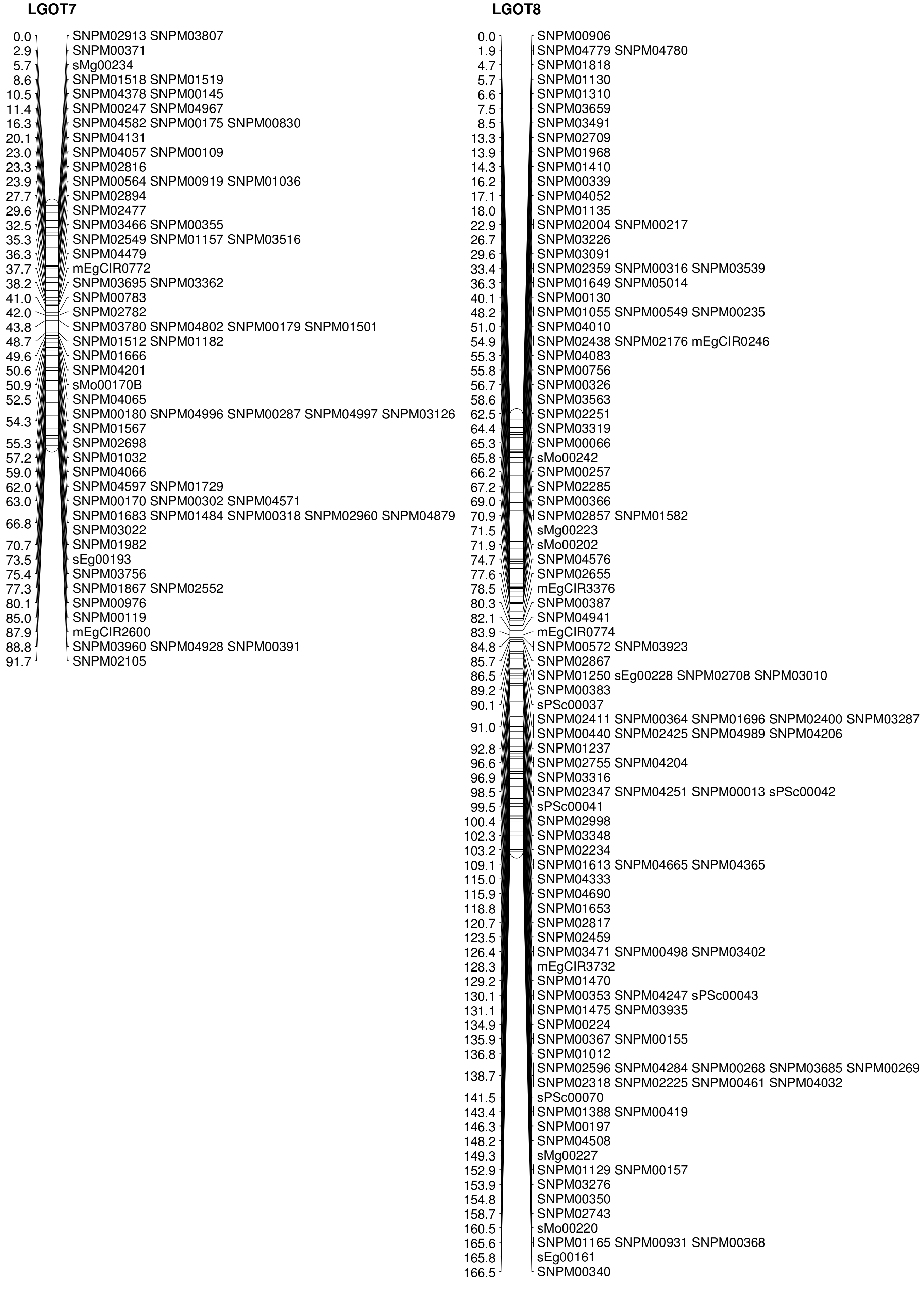
LGOT7
0.0 SNPM02913 SNPM03807
2.9 SNPM00371
5.7 sMg00234
8.6 SNPM01518 SNPM01519
10.5 SNPM04378 SNPM00145
11.4 SNPM00247 SNPM04967
16.3 SNPM04582 SNPM00175 SNPM00830
20.1 SNPM04131
23.0 SNPM04057 SNPM00109
23.3 SNPM02816
23.9 SNPM00564 SNPM00919 SNPM01036
27.7 SNPM02894
29.6 SNPM02477
32.5 SNPM03466 SNPM00355
35.3 SNPM02549 SNPM01157 SNPM03516
36.3 SNPM04479
37.7 mEgCIR0772
38.2 SNPM03695 SNPM03362
41.0 SNPM00783
42.0 SNPM02782
43.8 SNPM03780 SNPM04802 SNPM00179 SNPM01501
48.7 SNPM01512 SNPM01182
49.6 SNPM01666
50.6 SNPM04201
50.9 sMo00170B
52.5 SNPM04065
54.3 SNPM00180 SNPM04996 SNPM00287 SNPM04997 SNPM03126
SNPM01567
55.3 SNPM02698
57.2 SNPM01032
59.0 SNPM04066
62.0 SNPM04597 SNPM01729
63.0 SNPM00170 SNPM00302 SNPM04571
66.8 SNPM01683 SNPM01484 SNPM00318 SNPM02960 SNPM04879
SNPM03022
70.7 SNPM01982
73.5 sEg00193
75.4 SNPM03756
77.3 SNPM01867 SNPM02552
80.1 SNPM00976
85.0 SNPM00119
87.9 mEgCIR2600
88.8 SNPM03960 SNPM04928 SNPM00391
91.7 SNPM02105
LGOT8
0.0 SNPM00906
1.9 SNPM04779 SNPM04780
4.7 SNPM01818
5.7 SNPM01130
6.6 SNPM01310
7.5 SNPM03659
8.5 SNPM03491
13.3 SNPM02709
13.9 SNPM01968
14.3 SNPM01410
16.2 SNPM00339
17.1 SNPM04052
18.0 SNPM01135
22.9 SNPM02004 SNPM00217
26.7 SNPM03226
29.6 SNPM03091
33.4 SNPM02359 SNPM00316 SNPM03539
36.3 SNPM01649 SNPM05014
40.1 SNPM00130
48.2 SNPM01055 SNPM00549 SNPM00235
51.0 SNPM04010
54.9 SNPM02438 SNPM02176 mEgCIR0246
55.3 SNPM04083
55.8 SNPM00756
56.7 SNPM00326
58.6 SNPM03563
62.5 SNPM02251
64.4 SNPM03319
65.3 SNPM00066
65.8 sMo00242
66.2 SNPM00257
67.2 SNPM02285
69.0 SNPM00366
70.9 SNPM02857 SNPM01582
71.5 sMg00223
71.9 sMo00202
74.7 SNPM04576
77.6 SNPM02655
78.5 mEgCIR3376
80.3 SNPM00387
82.1 SNPM04941
83.9 mEgCIR0774
84.8 SNPM00572 SNPM03923
85.7 SNPM02867
86.5 SNPM01250 sEg00228 SNPM02708 SNPM03010
89.2 SNPM00383
90.1 sPSc00037
91.0 SNPM02411 SNPM00364 SNPM01696 SNPM02400 SNPM03287
SNPM00440 SNPM02425 SNPM04989 SNPM04206
92.8 SNPM01237
96.6 SNPM02755 SNPM04204
96.9 SNPM03316
98.5 SNPM02347 SNPM04251 SNPM00013 sPSc00042
99.5 sPSc00041
100.4 SNPM02998
102.3 SNPM03348
103.2 SNPM02234
109.1 SNPM01613 SNPM04665 SNPM04365
115.0 SNPM04333
115.9 SNPM04690
118.8 SNPM01653
120.7 SNPM02817
123.5 SNPM02459
126.4 SNPM03471 SNPM00498 SNPM03402
128.3 mEgCIR3732
129.2 SNPM01470
130.1 SNPM00353 SNPM04247 sPSc00043
131.1 SNPM01475 SNPM03935
134.9 SNPM00224
135.9 SNPM00367 SNPM00155
136.8 SNPM01012
138.7 SNPM02596 SNPM04284 SNPM00268 SNPM03685 SNPM00269
SNPM02318 SNPM02225 SNPM00461 SNPM04032
141.5 sPSc00070
143.4 SNPM01388 SNPM00419
146.3 SNPM00197
148.2 SNPM04508
149.3 sMg00227
152.9 SNPM01129 SNPM00157
153.9 SNPM03276
154.8 SNPM00350
158.7 SNPM02743
160.5 sMo00220
165.6 SNPM01165 SNPM00931 SNPM00368
165.8 sEg00161
166.5 SNPM00340

LGT9

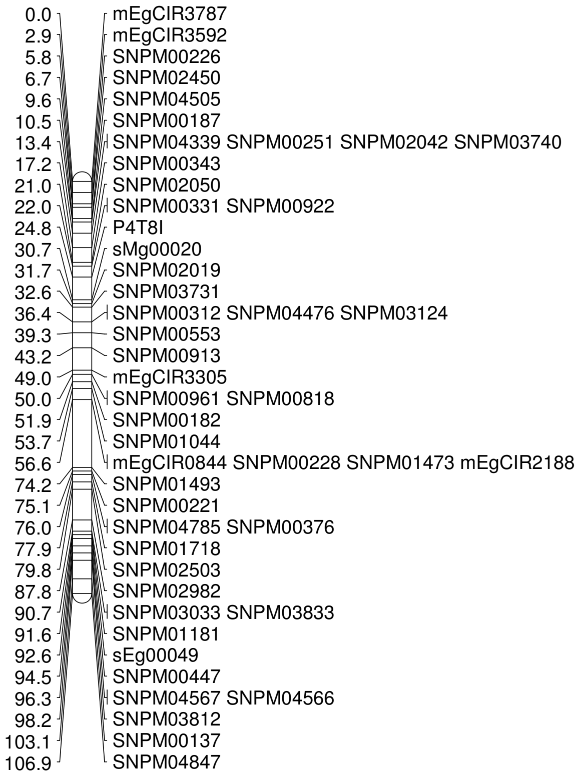

0.0 mEgCIR3787
2.9 mEgCIR3592
5.8 SNPM00226
6.7 SNPM02450
9.6 SNPM04505
10.5 SNPM00187
13.4 SNPM04339 SNPM00251 SNPM02042 SNPM03740
17.2 SNPM00343
21.0 SNPM02050
22.0 SNPM00331 SNPM00922
24.8 P4T8I
30.7 sMg00020
31.7 SNPM02019
32.6 SNPM03731
36.4 SNPM00312 SNPM04476 SNPM03124
39.3 SNPM00553
43.2 SNPM00913
49.0 mEgCIR3305
50.0 SNPM00961 SNPM00818
51.9 SNPM00182
53.7 SNPM01044
56.6 mEgCIR0844 SNPM00228 SNPM01473 mEgCIR2188
74.2 SNPM01493
75.1 SNPM00221
76.0 SNPM04785 SNPM00376
77.9 SNPM01718
79.8 SNPM02503
87.8 SNPM02982
90.7 SNPM03033 SNPM03833
91.6 SNPM01181
92.6 sEg00049
94.5 SNPM00447
96.3 SNPM04567 SNPM04566
98.2 SNPM03812
103.1 SNPM00137
106.9 SNPM04847

LGOT10

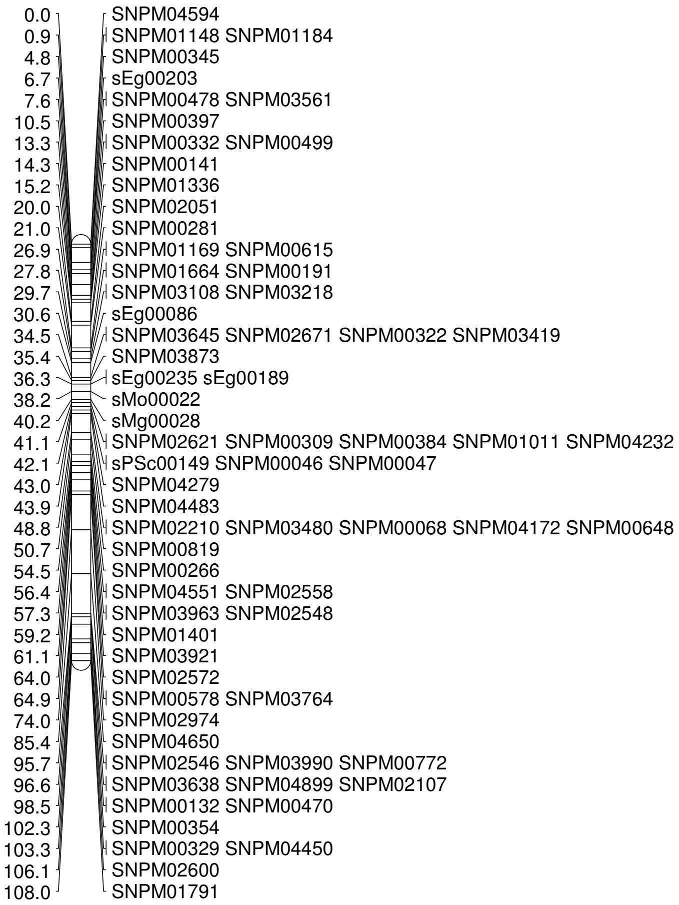

0.0 SNPM04594
0.9 SNPM01148 SNPM01184
4.8 SNPM00345
6.7 sEg00203
7.6 SNPM00478 SNPM03561
10.5 SNPM00397
13.3 SNPM00332 SNPM00499
14.3 SNPM00141
15.2 SNPM01336
20.0 SNPM02051
21.0 SNPM00281
26.9 SNPM01169 SNPM00615
27.8 SNPM01664 SNPM00191
29.7 SNPM03108 SNPM03218
30.6 sEg00086
34.5 SNPM03645 SNPM02671 SNPM00322 SNPM03419
35.4 SNPM03873
36.3 sEg00235 sEg00189
38.2 sMo00022
40.2 sMg00028
41.1 SNPM02621 SNPM00309 SNPM00384 SNPM01011 SNPM04232
42.1 sPSc00149 SNPM00046 SNPM00047
43.0 SNPM04279
43.9 SNPM04483
48.8 SNPM02210 SNPM03480 SNPM00068 SNPM04172 SNPM00648
50.7 SNPM00819
54.5 SNPM00266
56.4 SNPM04551 SNPM02558
57.3 SNPM03963 SNPM02548
59.2 SNPM01401
61.1 SNPM03921
64.0 SNPM02572
64.9 SNPM00578 SNPM03764
74.0 SNPM02974
85.4 SNPM04650
95.7 SNPM02546 SNPM03990 SNPM00772
96.6 SNPM03638 SNPM04899 SNPM02107
98.5 SNPM00132 SNPM00470
102.3 SNPM00354
103.3 SNPM00329 SNPM04450
106.1 SNPM02600
108.0 SNPM01791

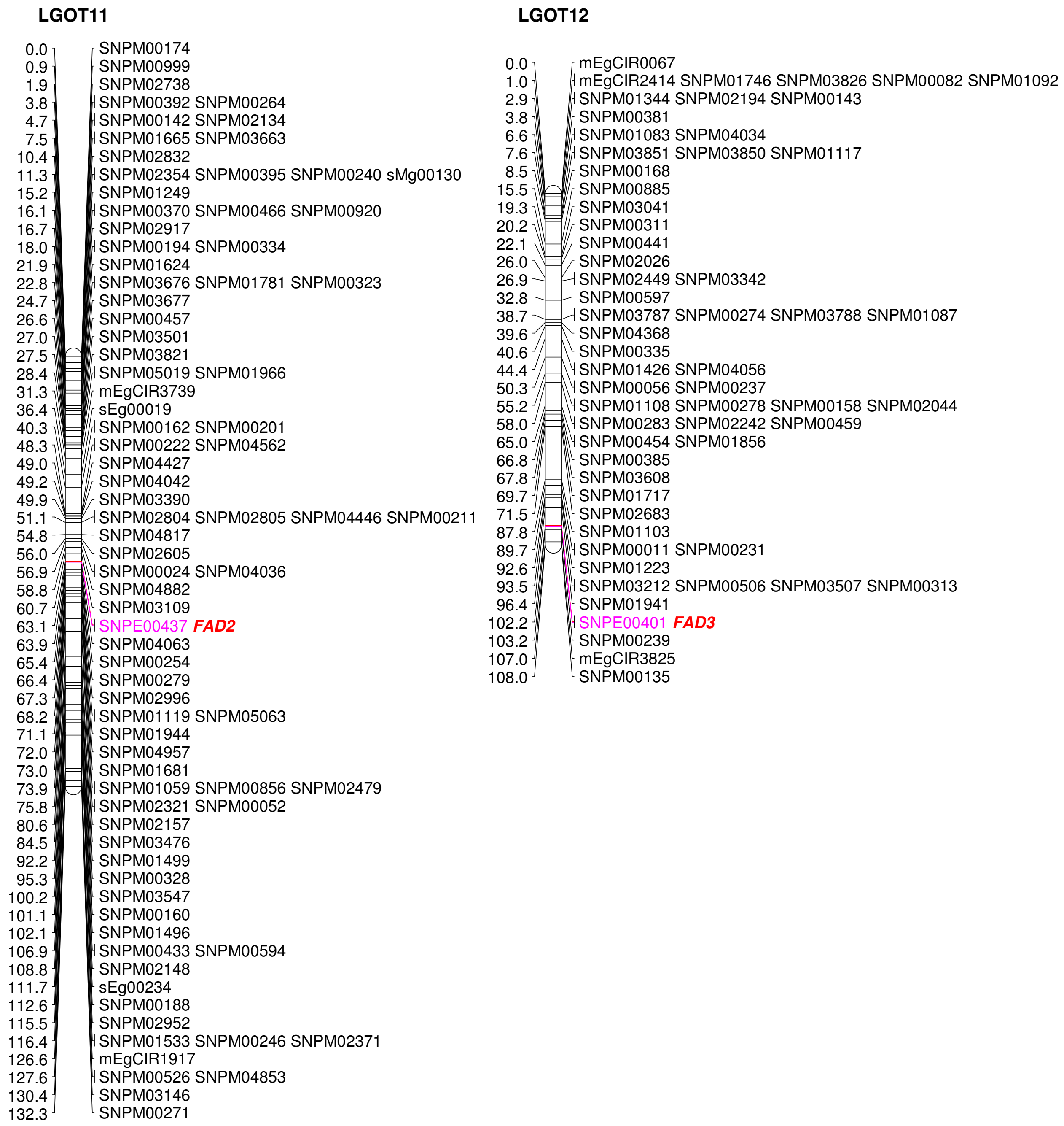

LGOT11
0.0 SNPM00174
0.9 SNPM00999
1.9 SNPM02738
3.8 SNPM00392 SNPM00264
4.7 SNPM00142 SNPM02134
7.5 SNPM01665 SNPM03663
10.4 SNPM02832
11.3 SNPM02354 SNPM00395 SNPM00240 sMg00130
15.2 SNPM01249
16.1 SNPM00370 SNPM00466 SNPM00920
16.7 SNPM02917
18.0 SNPM00194 SNPM00334
21.9 SNPM01624
22.8 SNPM03676 SNPM01781 SNPM00323
24.7 SNPM03677
26.6 SNPM00457
27.0 SNPM03501
27.5 SNPM03821
28.4 SNPM05019 SNPM01966
31.3 mEgCIR3739
36.4 sEg00019
40.3 SNPM00162 SNPM00201
48.3 SNPM00222 SNPM04562
49.0 SNPM04427
49.2 SNPM04042
49.9 SNPM03390
51.1 SNPM02804 SNPM02805 SNPM04446 SNPM00211
54.8 SNPM04817
56.0 SNPM02605
56.9 SNPM00024 SNPM04036
58.8 SNPM04882
60.7 SNPM03109
63.1 SNPE00437 FAD2
63.9 SNPM04063
65.4 SNPM00254
66.4 SNPM00279
67.3 SNPM02996
68.2 SNPM01119 SNPM05063
71.1 SNPM01944
72.0 SNPM04957
73.0 SNPM01681
73.9 SNPM01059 SNPM00856 SNPM02479
75.8 SNPM02321 SNPM00052
80.6 SNPM02157
84.5 SNPM03476
92.2 SNPM01499
95.3 SNPM00328
100.2 SNPM03547
101.1 SNPM00160
102.1 SNPM01496
106.9 SNPM00433 SNPM00594
108.8 SNPM02148
111.7 sEg00234
112.6 SNPM00188
115.5 SNPM02952
116.4 SNPM01533 SNPM00246 SNPM02371
126.6 mEgCIR1917
127.6 SNPM00526 SNPM04853
130.4 SNPM03146
132.3 SNPM00271
LGOT12
0.0 mEgCIR0067
1.0 mEgCIR2414 SNPM01746 SNPM03826 SNPM00082 SNPM01092
2.9 SNPM01344 SNPM02194 SNPM00143
3.8 SNPM00381
6.6 SNPM01083 SNPM04034
7.6 SNPM03851 SNPM03850 SNPM01117
8.5 SNPM00168
15.5 SNPM00885
19.3 SNPM03041
20.2 SNPM00311
22.1 SNPM00441
26.0 SNPM02026
26.9 SNPM02449 SNPM03342
32.8 SNPM00597
38.7 SNPM03787 SNPM00274 SNPM03788 SNPM01087
39.6 SNPM04368
40.6 SNPM00335
44.4 SNPM01426 SNPM04056
50.3 SNPM00056 SNPM00237
55.2 SNPM01108 SNPM00278 SNPM00158 SNPM02044
58.0 SNPM00283 SNPM02242 SNPM00459
65.0 SNPM00454 SNPM01856
66.8 SNPM00385
67.8 SNPM03608
69.7 SNPM01717
71.5 SNPM02683
87.8 SNPM01103
89.7 SNPM00011 SNPM00231
92.6 SNPM01223
93.5 SNPM03212 SNPM00506 SNPM03507 SNPM00313
96.4 SNPM01941
102.2 SNPE00401 FAD3
103.2 SNPM00239
107.0 mEgCIR3825
108.0 SNPM00135

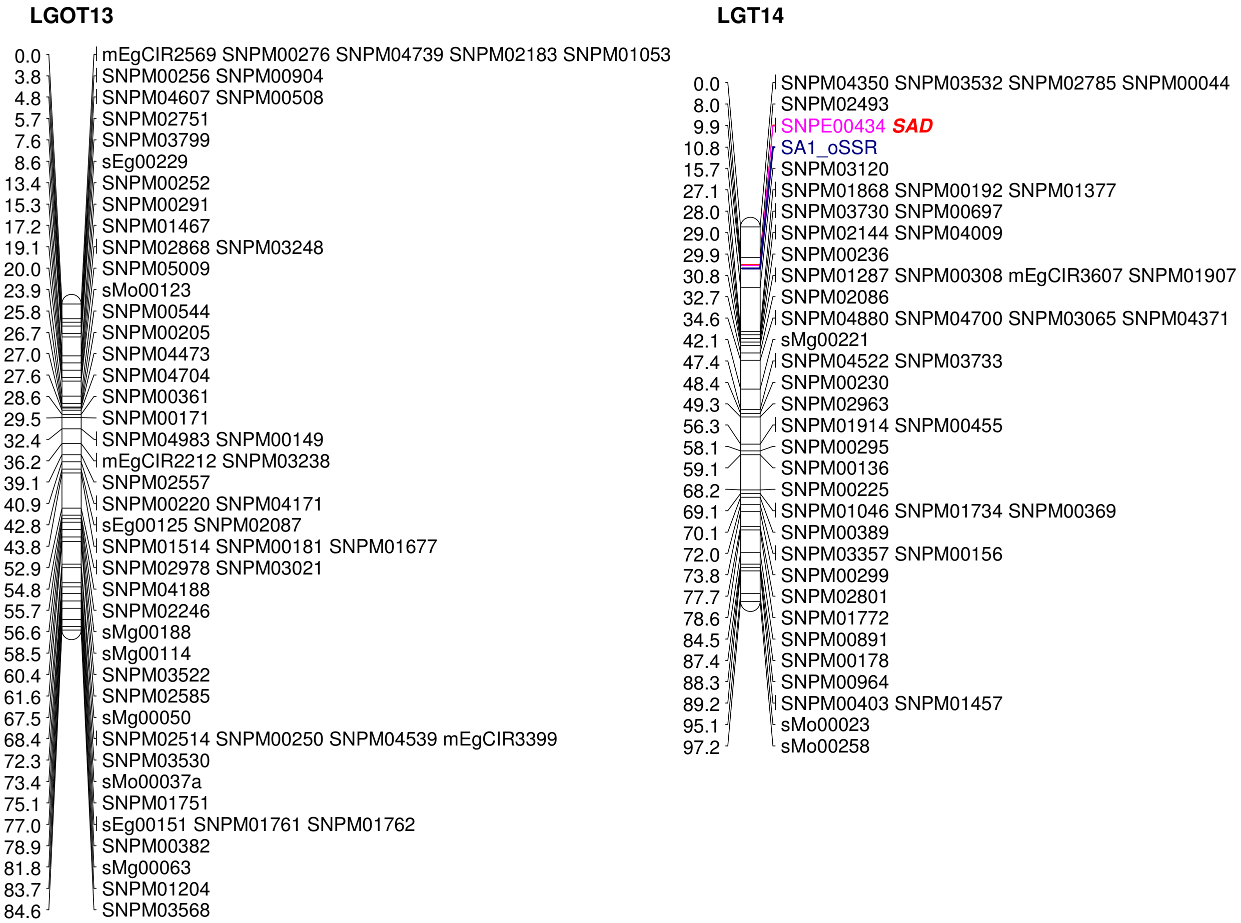
LGOT13
0.0 mEgCIR2569 SNPM00276 SNPM04739 SNPM02183 SNPM01053
3.8 SNPM00256 SNPM00904
4.8 SNPM04607 SNPM00508
5.7 SNPM02751
7.6 SNPM03799
8.6 sEg00229
13.4 SNPM00252
15.3 SNPM00291
17.2 SNPM01467
19.1 SNPM02868 SNPM03248
20.0 SNPM05009
23.9 sMo00123
25.8 SNPM00544
26.7 SNPM00205
27.0 SNPM04473
27.6 SNPM04704
28.6 SNPM00361
29.5 SNPM00171
32.4 SNPM04983 SNPM00149
36.2 mEgCIR2212 SNPM03238
39.1 SNPM02557
40.9 SNPM00220 SNPM04171
42.8 sEg00125 SNPM02087
43.8 SNPM01514 SNPM00181 SNPM01677
52.9 SNPM02978 SNPM03021
54.8 SNPM04188
55.7 SNPM02246
56.6 sMg00188
58.5 sMg00114
60.4 SNPM03522
61.6 SNPM02585
67.5 sMg00050
68.4 SNPM02514 SNPM00250 SNPM04539 mEgCIR3399
72.3 SNPM03530
73.4 sMo00037a
75.1 SNPM01751
77.0 sEg00151 SNPM01761 SNPM01762
78.9 SNPM00382
81.8 sMg00063
83.7 SNPM01204
84.6 SNPM03568
LGT14
0.0 SNPM04350 SNPM03532 SNPM02785 SNPM00044
8.0 SNPM02493
9.9 SNPE00434 SAD
10.8 SA1_oSSR
15.7 SNPM03120
27.1 SNPM01868 SNPM00192 SNPM01377
28.0 SNPM03730 SNPM00697
29.0 SNPM02144 SNPM04009
29.9 SNPM00236
30.8 SNPM01287 SNPM00308 mEgCIR3607 SNPM01907
32.7 SNPM02086
34.6 SNPM04880 SNPM04700 SNPM03065 SNPM04371
42.1 sMg00221
47.4 SNPM04522 SNPM03733
48.4 SNPM00230
49.3 SNPM02963
56.3 SNPM01914 SNPM00455
58.1 SNPM00295
59.1 SNPM00136
68.2 SNPM00225
69.1 SNPM01046 SNPM01734 SNPM00369
70.1 SNPM00389
72.0 SNPM03357 SNPM00156
73.8 SNPM00299
77.7 SNPM02801
78.6 SNPM01772
84.5 SNPM00891
87.4 SNPM00178
88.3 SNPM00964
89.2 SNPM00403 SNPM01457
95.1 sMo00023
97.2 sMo00258

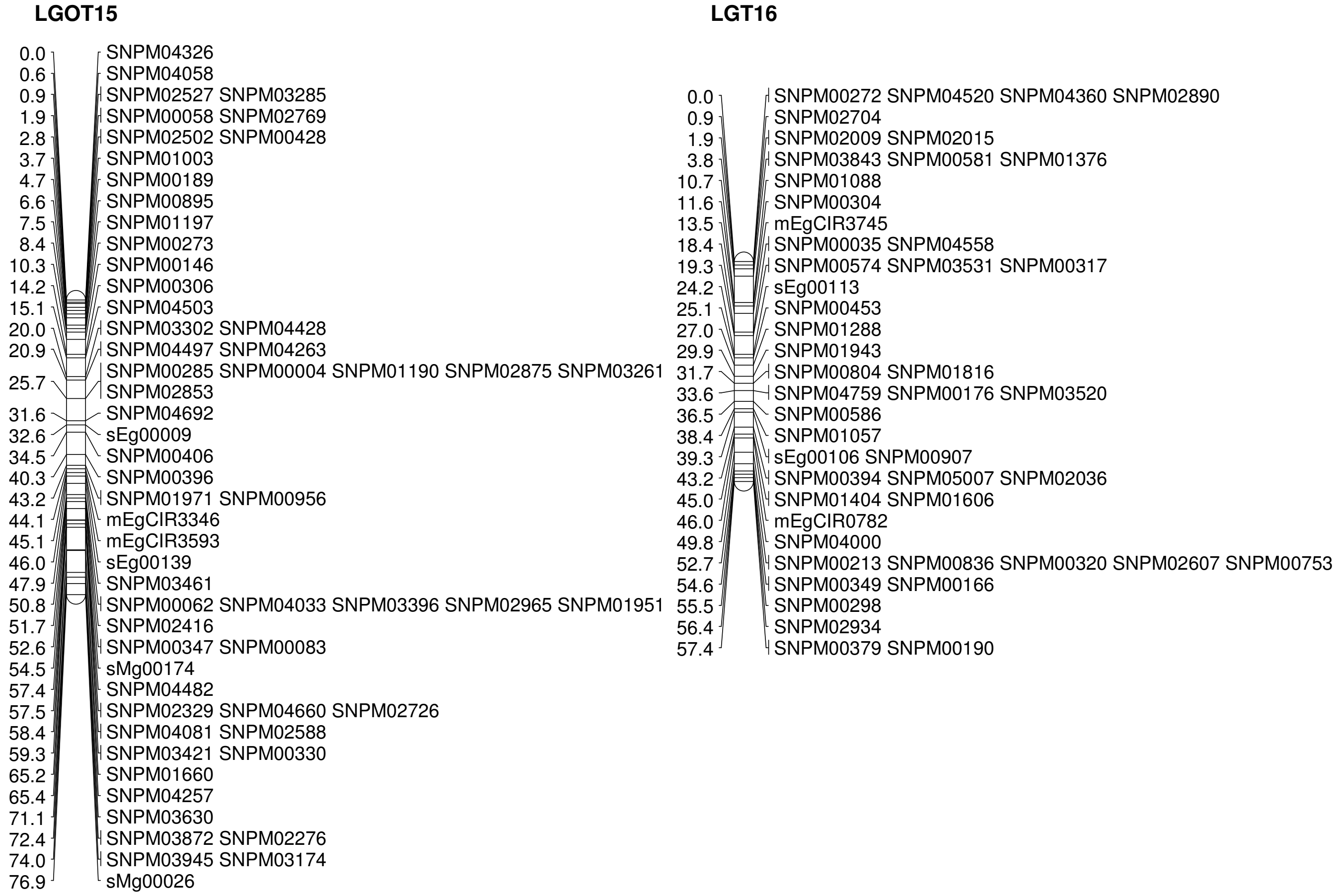
LGOT15
0.0 SNPM04326
0.6 SNPM04058
0.9 SNPM02527 SNPM03285
1.9 SNPM00058 SNPM02769
2.8 SNPM02502 SNPM00428
3.7 SNPM01003
4.7 SNPM00189
6.6 SNPM00895
7.5 SNPM01197
8.4 SNPM00273
10.3 SNPM00146
14.2 SNPM00306
15.1 SNPM04503
20.0 SNPM03302 SNPM04428
20.9 SNPM04497 SNPM04263
25.7 SNPM00285 SNPM00004 SNPM01190 SNPM02875 SNPM03261
SNPM02853
31.6 SNPM04692
32.6 sEg00009
34.5 SNPM00406
40.3 SNPM00396
43.2 SNPM01971 SNPM00956
44.1 mEgCIR3346
45.1 mEgCIR3593
46.0 sEg00139
47.9 SNPM03461
50.8 SNPM00062 SNPM04033 SNPM03396 SNPM02965 SNPM01951
51.7 SNPM02416
52.6 SNPM00347 SNPM00083
54.5 sMg00174
57.4 SNPM04482
57.5 SNPM02329 SNPM04660 SNPM02726
58.4 SNPM04081 SNPM02588
59.3 SNPM03421 SNPM00330
65.2 SNPM01660
65.4 SNPM04257
71.1 SNPM03630
72.4 SNPM03872 SNPM02276
74.0 SNPM03945 SNPM03174
76.9 sMg00026
LGT16
0.0 SNPM00272 SNPM04520 SNPM04360 SNPM02890
0.9 SNPM02704
1.9 SNPM02009 SNPM02015
3.8 SNPM03843 SNPM00581 SNPM01376
10.7 SNPM01088
11.6 SNPM00304
13.5 mEgCIR3745
18.4 SNPM00035 SNPM04558
19.3 SNPM00574 SNPM03531 SNPM00317
24.2 sEg00113
25.1 SNPM00453
27.0 SNPM01288
29.9 SNPM01943
31.7 SNPM00804 SNPM01816
33.6 SNPM04759 SNPM00176 SNPM03520
36.5 SNPM00586
38.4 SNPM01057
39.3 sEg00106 SNPM00907
43.2 SNPM00394 SNPM05007 SNPM02036
45.0 SNPM01404 SNPM01606
46.0 mEgCIR0782
49.8 SNPM04000
52.7 SNPM00213 SNPM00836 SNPM00320 SNPM02607 SNPM00753
54.6 SNPM00349 SNPM00166
55.5 SNPM00298
56.4 SNPM02934
57.4 SNPM00379 SNPM00190
