# Supplementary material for: Fine-mapping and cross-validation of QTLs linked to fatty acid composition in multiple independent interspecific crosses of oil palm
Source: BMC Genomics. 2016 Apr 14;17:289. doi: 10.1186/s12864-016-2607-4 (PMC4832457; doi:10.1186/s12864-016-2607-4)
Supplement: Additional file 2: — Pearson’s correlation coefficients for iodine value (IV) and fatty acid composition (FAC) in palm oil of OxG mapping population. (PDF 13 kb) [file 12864_2016_2607_MOESM2_ESM.pdf]

Trait	C14:0	C16:0	C16:1	Lg_{10}C18:0	C18:1	C18:2	C18:3
**IV**	-0.651**	-0.864**	-0.039	-0.217*	0.740**	0.449**	0.169
**C14:0**		0.699**	0.178	-0.077	-0.644**	-0.279**	0.170
**C16:0**			0.089	0.113	-0.949**	-0.162	-0.094
**C16:1**				-0.744**	-0.141	0.067	0.289**
**Lg_{10}C18:0**					-0.073	-0.192	-0.288**
**C18:1**						-0.098	-0.025
**C18:2**							0.228*

* Correlation significant at 0.05 level (2-tailed)

**Correlation significant at 0.01 level (2-tailed)
