# Supplementary material for: Fine-mapping and cross-validation of QTLs linked to fatty acid composition in multiple independent interspecific crosses of oil palm
Source: BMC Genomics. 2016 Apr 14;17:289. doi: 10.1186/s12864-016-2607-4 (PMC4832457; doi:10.1186/s12864-016-2607-4)
Supplement: Additional file 3: — Pearson’s correlation coefficients for iodine value (IV) and fatty acid composition (FAC) in palm oil of BC2 (2.6-1) validation cross. (PDF 13 kb) [file 12864_2016_2607_MOESM3_ESM.pdf]

Trait	C14:0	C16:0	C16:1	C18:0	C18:1	C18:2	C18:3
**$Lg_{10}IV$**	-0.741**	-0.752**	-0.139	-0.221	0.509**	0.507**	0.339*
**C14:0**		0.718**	0.293*	-0.111	-0.417**	-0.382**	-0.099
**C16:0**			0.423**	-0.261	-0.745**	-0.029	-0.097
**C16:1**				-0.662**	-0.041	-0.108	0.000
**C18:0**					-0.236	0.030	-0.225
**C18:1**						-0.482**	0.027
**C18:2**							0.257

* Correlation significant at 0.05 level (2-tailed)

**Correlation significant at 0.01 level (2-tailed)
