# Supplementary material for: Fine-mapping and cross-validation of QTLs linked to fatty acid composition in multiple independent interspecific crosses of oil palm
Source: BMC Genomics. 2016 Apr 14;17:289. doi: 10.1186/s12864-016-2607-4 (PMC4832457; doi:10.1186/s12864-016-2607-4)
Supplement: Additional file 4: — Pearson’s correlation coefficients for iodine value (IV) and fatty acid composition (FAC) in palm oil of BC2 (2.6-5) validation cross. (PDF 13 kb) [file 12864_2016_2607_MOESM4_ESM.pdf]

Trait	C14:0	C16:0	C16:1	C18:0	C18:1	C18:2	C18:3
**IV**	-0.289*	-0.709**	0.064	0.177	0.191	0.546**	0.223
**C14:0**		0.484**	0.421**	0.050	-0.517**	0.054	0.280*
**C16:0**			0.213	-0.318*	-0.704**	-0.036	-0.059
**C16:1**				-0.400**	-0.130	-0.025	0.468**
**C18:0**					-0.171	0.262*	0.259
**C18:1**						-0.629**	-0.218
**C18:2**							0.180

* Correlation significant at 0.05 level (2-tailed)

**Correlation significant at 0.01 level (2-tailed)
