# Supplementary figures and images for: Fine-mapping and cross-validation of QTLs linked to fatty acid composition in multiple independent interspecific crosses of oil palm
Source: BMC Genomics. 2016 Apr 14;17:289. doi: 10.1186/s12864-016-2607-4 (PMC4832457; doi:10.1186/s12864-016-2607-4)

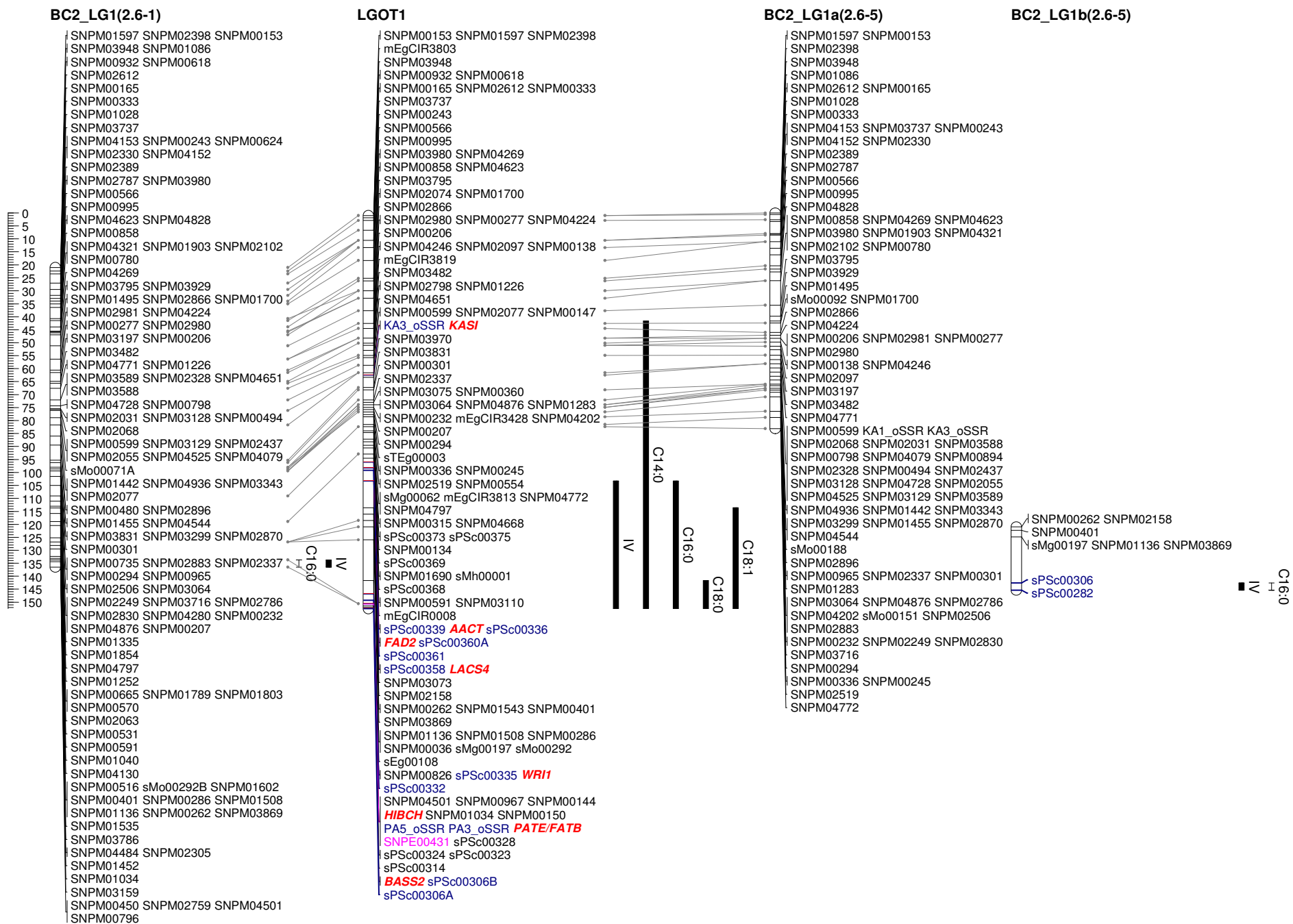

# BC2\_LG2(2.6-1)

# LGT2

# BC2\_LG2(2.6-5)

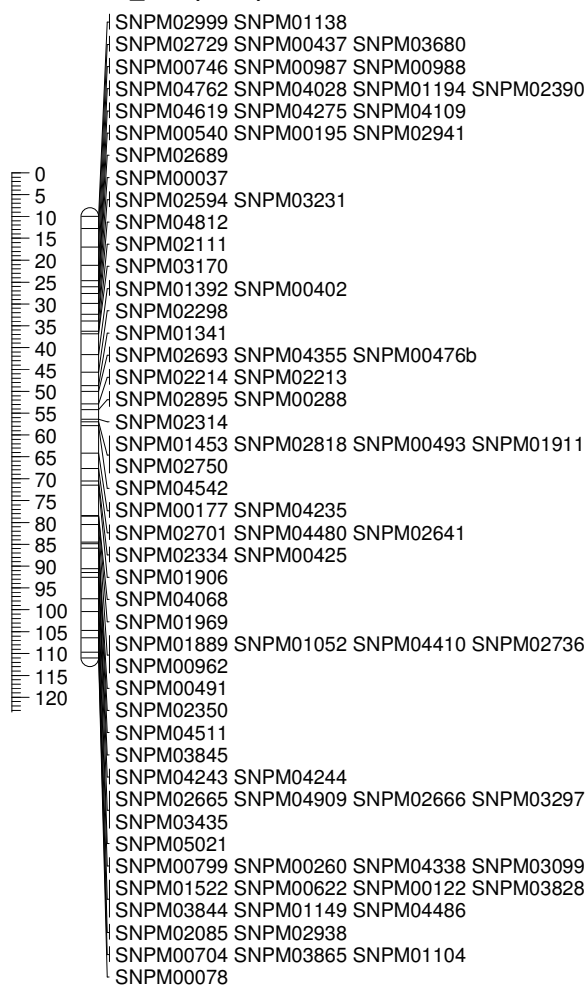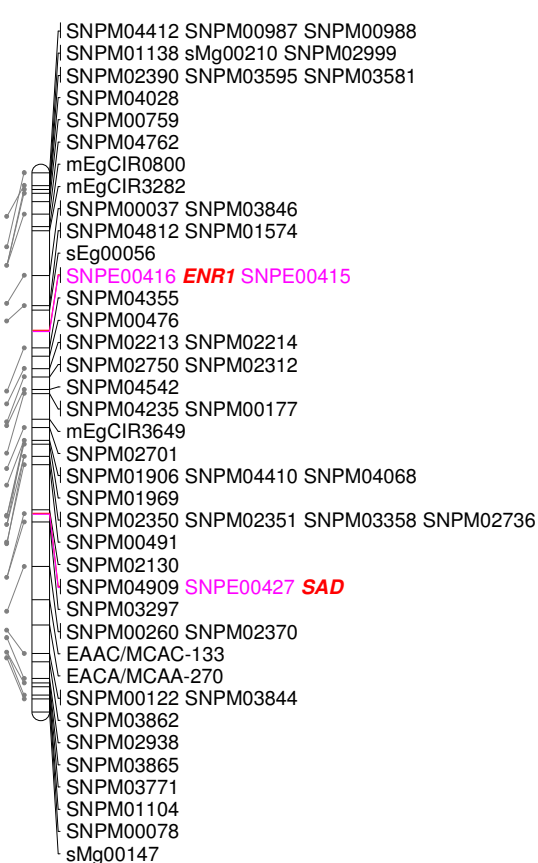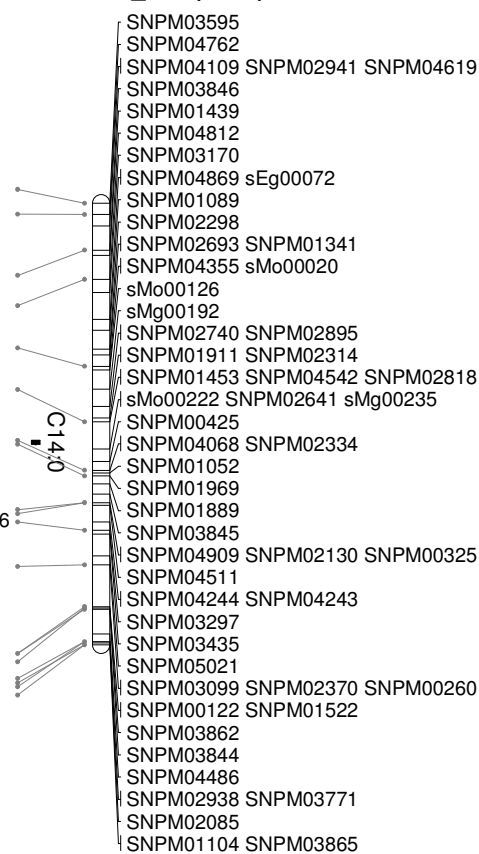

# BC2\_LG3(2.6-1)

# LGT3

# BC2\_LG3(2.6-5)

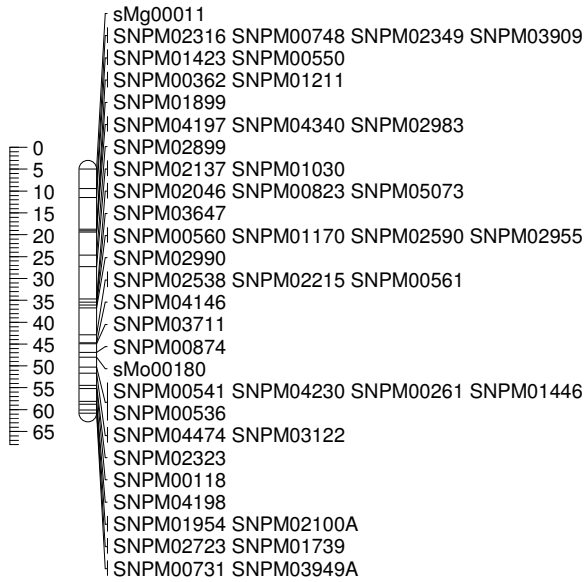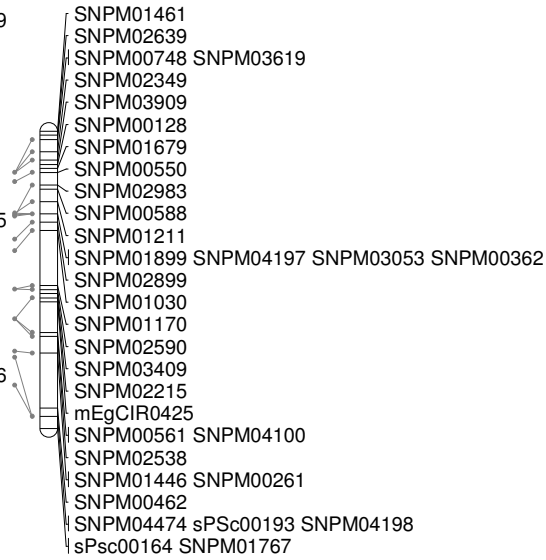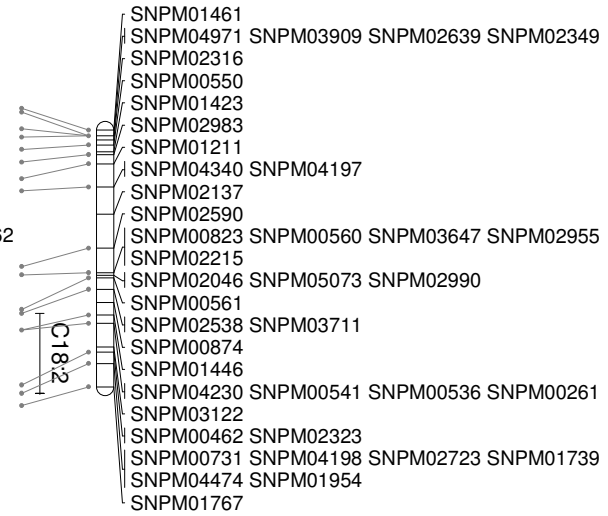



## BC2\_LG4(2.6-1)

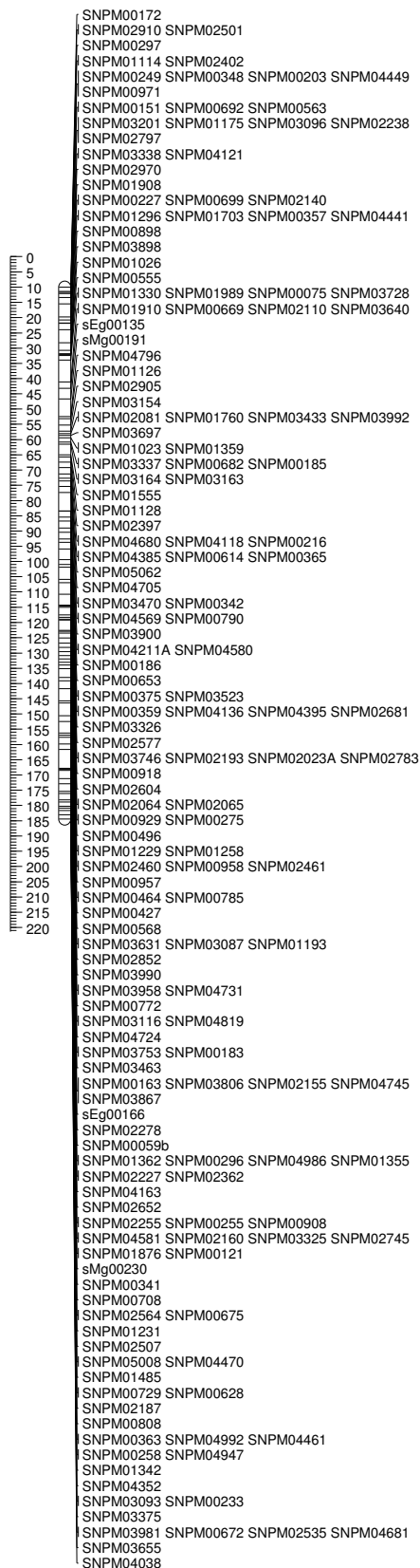

## LGOT4

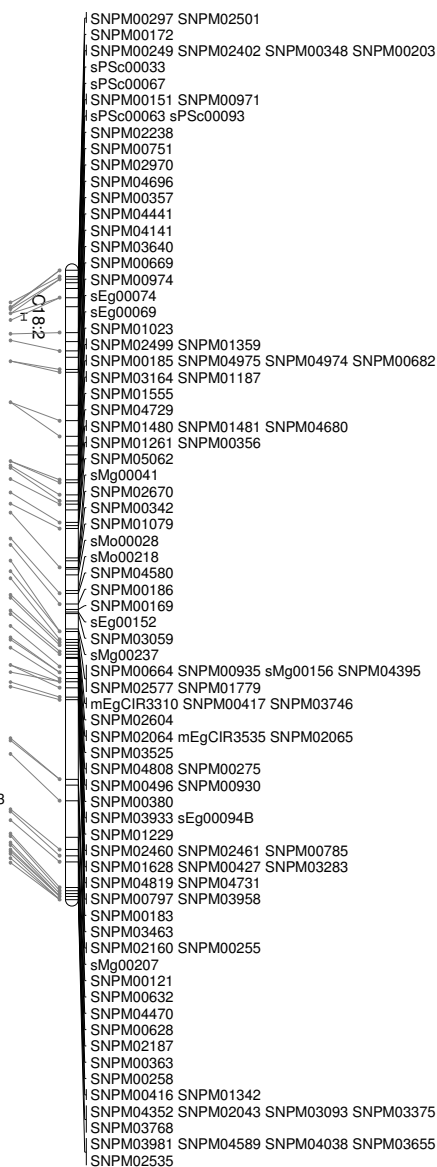

## BC2\_LG4(2.6-5)

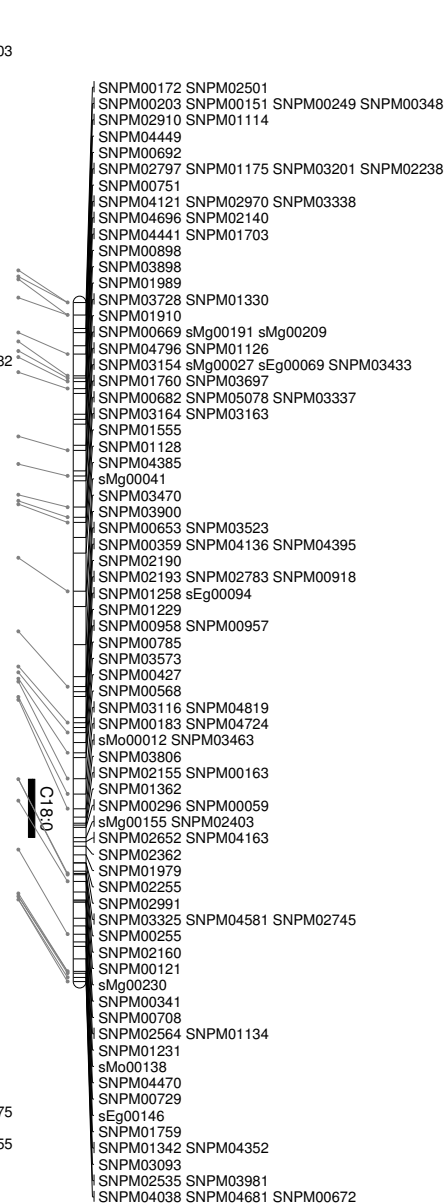

## BC2\_LG6(2.6-1)

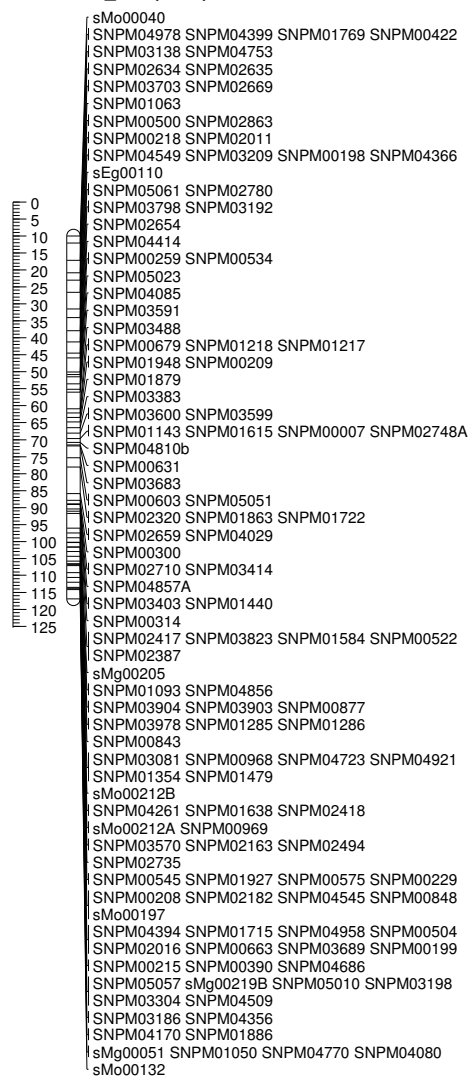

## LGOT6

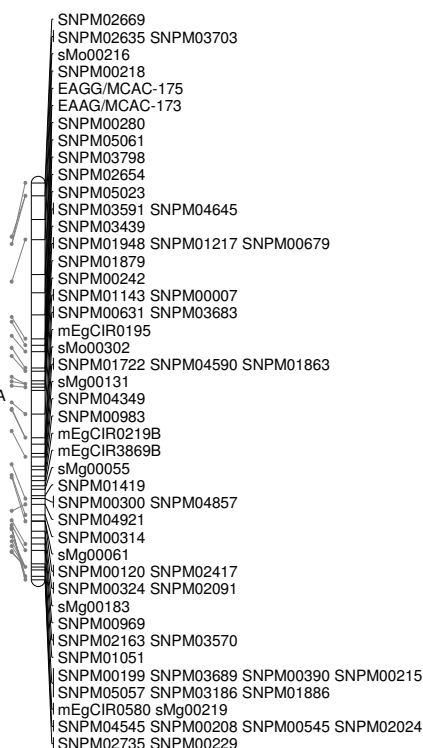

## BC2\_LG6(2.6-5)

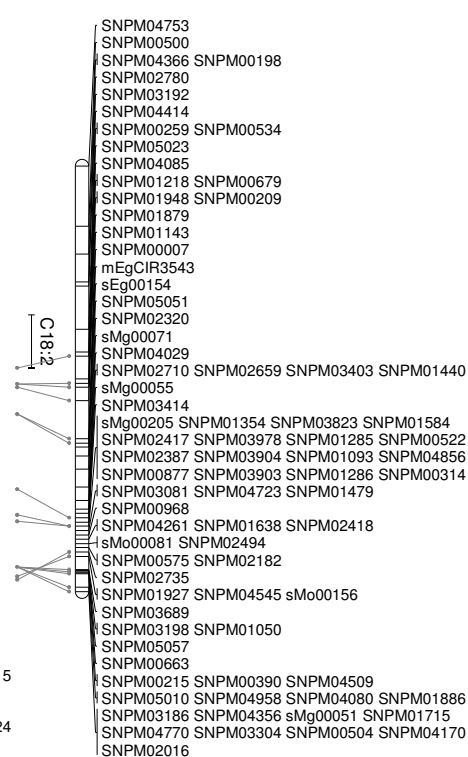

# BC2\_LG9(2.6-1)

# LGT9

# BC2\_LG9(2.6-5)

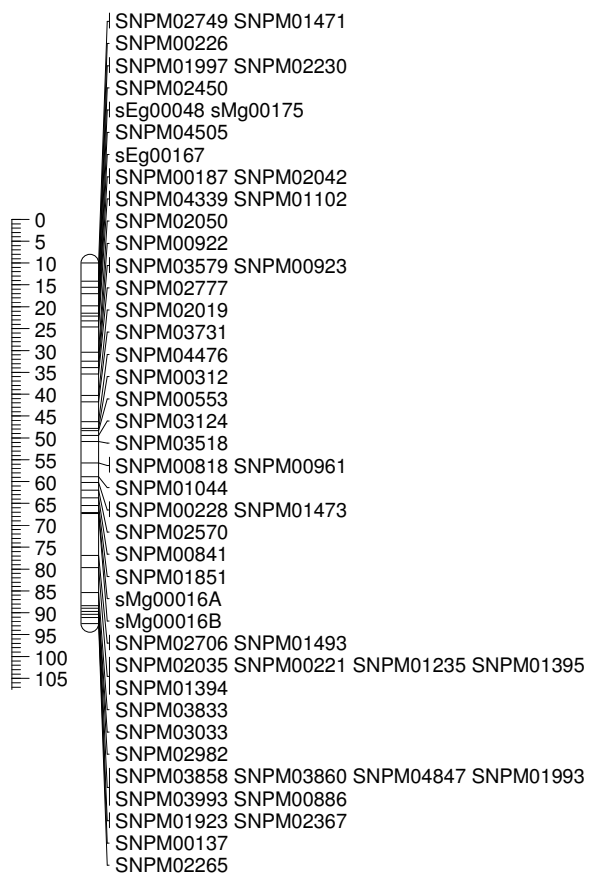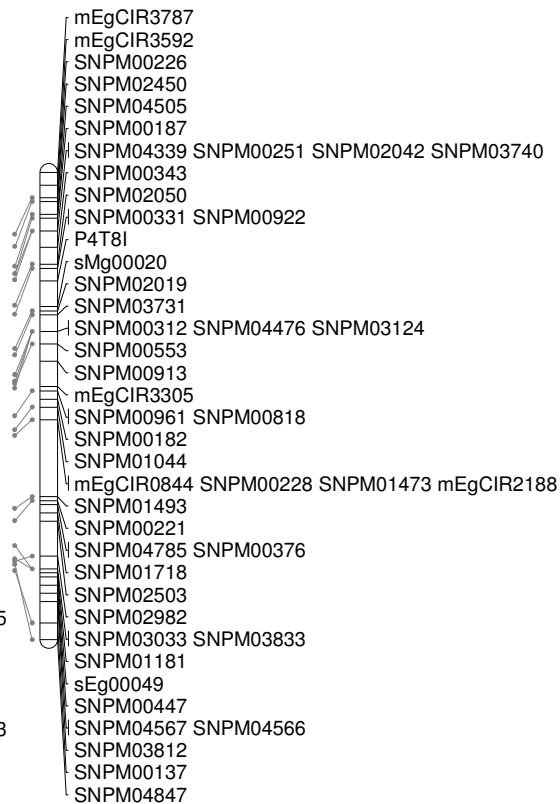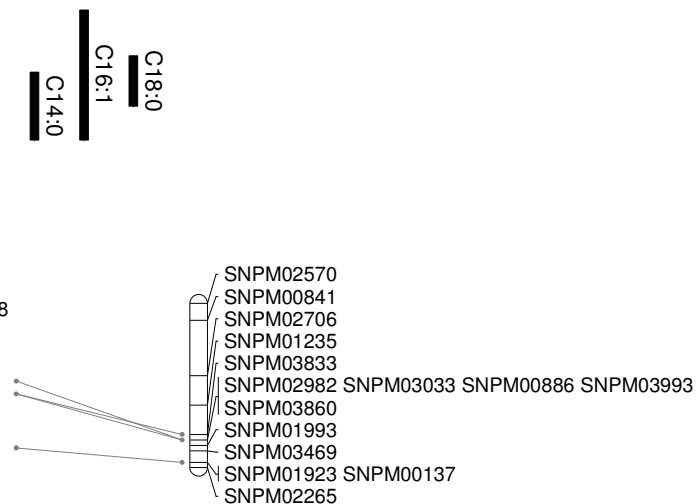

Supplement: Additional file 5: — Co-linearity of markers between the OxG and BC2 (2.6-1 & 2.6-5) populations as observed in six linkage groups hosting QTLs (OT1, T2, T3, OT4, OT6 and T9). (PDF 96 kb) [file 12864_2016_2607_MOESM5_ESM.pdf]
